# A Comparison of Pathophysiology in Humans and Rodent Models of Subarachnoid Hemorrhage

**DOI:** 10.3389/fnmol.2018.00071

**Published:** 2018-03-22

**Authors:** Jenna L. Leclerc, Joshua M. Garcia, Matthew A. Diller, Anne-Marie Carpenter, Pradip K. Kamat, Brian L. Hoh, Sylvain Doré

**Affiliations:** ^1^Department of Anesthesiology, University of Florida, Gainesville, FL, United States; ^2^Department of Neuroscience, Center for Translational Research in Neurodegenerative Disease, McKnight Brain Institute, University of Florida, Gainesville, FL, United States; ^3^Department of Neurosurgery, University of Florida, Gainesville, FL, United States; ^4^Department of Neurology, Psychiatry, and Pharmaceutics, University of Florida, Gainesville, FL, United States

**Keywords:** aneurysm, heme, hemoglobin, ischemia, iron, stroke, vasospasm

## Abstract

Non-traumatic subarachnoid hemorrhage (SAH) affects an estimated 30,000 people each year in the United States, with an overall mortality of ~30%. Most cases of SAH result from a ruptured intracranial aneurysm, require long hospital stays, and result in significant disability and high fatality. Early brain injury (EBI) and delayed cerebral vasospasm (CV) have been implicated as leading causes of morbidity and mortality in these patients, necessitating intense focus on developing preclinical animal models that replicate clinical SAH complete with delayed CV. Despite the variety of animal models currently available, translation of findings from rodent models to clinical trials has proven especially difficult. While the explanation for this lack of translation is unclear, possibilities include the lack of standardized practices and poor replication of human pathophysiology, such as delayed cerebral vasospasm and ischemia, in rodent models of SAH. In this review, we summarize the different approaches to simulating SAH in rodents, in particular elucidating the key pathophysiology of the various methods and models. Ultimately, we suggest the development of standardized model of rodent SAH that better replicates human pathophysiology for moving forward with translational research.

## Introduction

Although a variety of rodent models for subarachnoid hemorrhage (SAH) are in use, there is no standardized method of simulating the human equivalent, making translation to clinical observations challenging. Development of a rodent model began with Barry et al., who induced SAH by puncturing the basilar artery with a tungsten electrode (Barry et al., 1979). Additional models have been developed, predominantly involving intravascular perforation of a vessel in the Circle of Willis or direct injection of blood into the cisterna magna or prechiasmatic cistern. While each of these models has their advantages and disadvantages, none involve the spontaneous rupture of an intracranial aneurysm, as is observed in the majority of clinical cases. Thus, the purpose of this review is to address the current data surrounding SAH models and subsequently propose a bridge between these models and those that would more replicate the human equivalent to direct future preclinical model development and clinical studies.

## Clinical SAH

Non-traumatic SAH affects ~30,000 people per year in the United States (Rincon et al., 2013), with ~15% of patients dying before they ever reach the hospital (Connolly et al., 2012) and in-hospital mortality estimated at 20% (Rincon et al., 2013). Around 10% of SAH cases are due to non-aneurysmal bleeding in idiopathic perimesencephalic hemorrhage, while another 5% are due to anomalies such as intracranial arterial dissections and vascular malformations, among other rare causes (Marder et al., 2014). The remaining 85% of clinical SAH results from the spontaneous rupture of a cerebral aneurysm (Van Gijn and Rinkel, 2001). Intracranial aneurysms form at sites of high shear wall stress such as the arterial bifurcations in the Circle of Willis (Wong et al., 2008). The most common sites include the anterior cerebral artery (ACA), internal carotid artery (ICA), or the middle cerebral artery (MCA); whereas, aneurysms in vessels of the posterior circulation are less frequent but routinely observed (Wong et al., 2008). Symptom onset is characteristically marked by a sudden headache, often described as “the worst headache of my life (Gorelick et al., 1986).” As the majority of cases are due to aneurysmal subarachnoid hemorrhage (aSAH) and because non-aneurysmal SAH patients tend to experience fewer complications and better outcomes than aSAH patients (Cánovas et al., 2012; Boswell et al., 2013), the majority of this review will focus on aSAH.

Most patients surviving the initial bleed are critically ill and require prolonged intensive care unit stay (Diringer, 2009), resulting in significant public health costs. Additionally, aSAH has an earlier mean age of onset and is associated with higher disability and morbidity rates when compared to other types of stroke (Kolias et al., 2009). It has been shown that early treatment of aSAH increases the likelihood of having no to minimal disability following discharge from the hospital (Siddiq et al., 2012). Thus, it is important to understand the pathophysiology of aSAH in order to ensure its early treatment and direct preclinical studies to expound on existing standards of care.

### Cerebral vasospasm and delayed cerebral ischemia in clinical SAH

Following aSAH, patients often develop complications from the bleed that contribute to the high mortality rate of this disease. Hydrocephalus, seizures, cerebral ischemia, tissue shifts and herniations, hyponatremia, cardiac anomalies, and respiratory depression are formidable consequences that can result (Diringer et al., 2011). However, the leading cause of morbidity and mortality after aSAH is delayed cerebral ischemia (DCI).

DCI occurs in nearly 33% of aSAH cases and is defined as new focal neurological signs, acute mental status decline, or appearance of new infarction on computed tomography or magnetic resonance imaging (MRI) (MacDonald et al., 2014). Clinical identification of DCI is often difficult since fever, infection, hypoxia, sedatives, and electrolyte imbalances produce a similar clinical picture (Vergouwen et al., 2010). Additionally, acute mental status decline is undetectable in the subset of SAH patients that remain comatose throughout hospitalization; thus, the incidence of DCI may be higher than documented. Further work to elucidate the underlying mechanism of DCI will allow for development of additional treatments that may prove more effective.

The most supported theory regarding the pathogenesis of DCI points to a phenomenon known as cerebral vasospasm (CV), which is a narrowing of cerebral arteries leading to a transiently sustained interruption of blood flow to the brain parenchyma (Velat et al., 2011). Approximately 30–70% of aSAH patients, will experience CV between days 4 and 14 after aneurysm rupture, with peak vessel constriction occurring on days 7 and 8 (Izzy and Muehlschlegel, 2014), making the identification, treatment, and prevention of CV paramount to achieving favorable outcomes following aSAH. Because CV is a causative mechanism of DCI, treatment of diagnosed DCI focuses on attempting to reverse CV by inducing hypertension, hypervolumia, and hemodilution (HHH therapy)(Siasios et al., 2013). Although such measures can be helpful after symptom onset, it nevertheless remains an enigma why certain patients develop CV and symptomatic ischemia following aSAH, while others remain asymptomatic with minimal CV. Currently, the only documented and verified risk factor for the development of CV is a larger hemorrhage volume assessed by CT scan and quantitated using the Fisher scoring system (Fisher et al., 1980; Ko et al., 2016).

### Pathophysiology of clinical SAH

After aneurysm rupture, blood enters the subarachnoid space at arterial pressure and produces immediate pathophysiological effects and early brain injury (EBI) (MacDonald et al., 2014). Intracranial pressure (ICP) rises above 20 mmHg, mean arterial blood pressure (MABP) falls reflexively, and cerebral perfusion pressure (CPP) is reduced; this can lead to severe headache or syncope due to decreased cerebral blood flow (CBF) (Voldby and Enevoldsen, 1982). Following acute conditions, vessel constriction due to delayed CV contributes to further reductions in CPP (Dhar et al., 2012). In practice, detecting impairment in CPP early in the management of SAH is essential in monitoring for DCI (Diringer et al., 2011). Ischemia, infarction, hydrocephalus, and impaired cerebral autoregulation further contribute to increased ICP and exacerbate the reductions in CPP and CBF (Zoerle et al., 2015). A delicate balance must be maintained with managing MABP following SAH, as increases can lead to elevation of ICP, while decreases may result in further worsening of CPP and exacerbation of DCI. Current recommendations for MABP focus on hemodynamic stability, encouraging a stepwise titration of MABP with assessment of neurological status at each level to determine if the target value is appropriate (Diringer et al., 2011). Overall, vigilant management of physiological outcomes is critical in the SAH patient, as extremes in ICP, CPP, CBF, and MABP can ultimately lead to poor functional outcomes (Zoerle et al., 2015).

### Mortality and functional outcomes of clinical SAH

After aSAH, up to 15% of patients will die immediately following the ictus, and the total case fatality rate approaches 50% (MacDonald et al., 2014). Long-term survival is correlated with increased consciousness and neurological grade on admission, less blood volume on initial CT scan, and age at ictus (Rosengart et al., 2007). Clinically, stratifying SAH based on the acute presentation can be accomplished with several widely used rating systems, such as the Glasgow Coma Scale, World Federation of Neurological Surgeon's scale, Fisher grade, and Hunt and Hess scale. These scales each have their own utility and are aimed at predicting the risk for CV or clinical outcome based on groupings of symptoms such as amount of subarachnoid blood, degree of mentation, focal deficits, and motor dysfunction. These scales integrate information regarding risk factors and symptoms in an effort to guide management and predict prognosis. In addition to stratification of SAH presentations, functional assessment tools are widely used for survivors of the initial hemorrhage. These instruments assign a quantitative value to deficits following SAH, analyzing factors such as language and speech, motor function and sensory loss, consciousness, coordination, and independence in activities of daily living (McGeoch et al., 2002; Rademaker et al., 2002a,c,d). Measures include the National Institutes of Health Stroke Scale (NIHSS), Barthel Index, modified Rankin scale (mRS), and Glasgow Outcome Scale (GOS).

Those who survive aSAH experience long-term complications such as memory impairment, epilepsy, neurocognitive dysfunction, neuropsychiatric disturbances, and focal neurological deficits (Al-Khindi et al., 2010). Hütter et al. (1995) published that SAH patients report deficits in verbal short-term memory, concentration, language, motivation, interests, mental capacity, free-time activities, social relationships, and fine motor coordination (Hütter et al., 1995). Longitudinally, 40–50% of patients require help in common household activities, and almost 50% exhibit disability in leisure and vocational activities (Lindberg et al., 1992). Approximately 40% will be cognitively impaired (Dombovy et al., 1998), which is influenced by the incidence of CV, DCI, and infarction, but unrelated to the initial location of the ruptured aneurysm (MacDonald et al., 2012). Cognitive domain deficits commonly affected in aSAH patients with DCI are verbal memory, language, and visuospatial memory and skills (Caeiro et al., 2011; Chu et al., 2015a). DCI has been associated with poor outcomes after SAH, but even with good outcomes, persistent cognitive deficits can still manifest, limiting psychosocial functioning. The correlation between neuropsychological and neurophysiological measures indicate frontal lobe damage, which in some patients persisted for years after the initial insult (Ravnik et al., 2006). Additionally, cognitive deficits also occurs in patients with CV and no DCI (Larsson et al., 1989; Richardson, 1991; Pluta et al., 2009; Miller et al., 2014). Neuropsychiatric disturbances including depression, anxiety, apathy, and sleep disorders are common following aSAH (Hackett and Anderson, 2000). Patients that do not undergo neuropsychological testing and subsequent treatment following SAH have worse outcomes than those that do (Kreiter et al., 2002), indicating a pressing need to evaluate all SAH patients for potential cognitive disability. It has also been shown that the Glascow Coma Scale score is able to predict self-reported quality of life in patients, but is otherwise unable to predict neurocognitive impairment (Cedzich and Roth, 2005). Little is known about the molecular pathways involved in mediating these long-term neurocognitive and neuropsychiatric outcomes after SAH. These findings indicate a need for additional mechanistic research and more efficient tools to predict functional outcomes, especially neurocognitive and neuropsychiatric impairments in SAH patients.

SAH is a devastating clinical disease with numerous debilitating outcomes. Understanding EBI, the pathophysiological changes that occur, and identifying predictors of CV and DCI will improve functional outcomes and reduce mortality following SAH. In order to further improve the outcomes of SAH patients, a standardized rodent model that better replicates human pathophysiology must be developed for use in preclinical studies.

## Preclinical models of non-aneurysmal SAH

While most cases of SAH in humans are due to rupture of an intracranial aneurysm, the majority of rodent studies have used models that more mimic non-aneurysmal SAH. This disparity is most likely due to the difficulty in producing a cerebral aneurysm in rodents (Hashimoto et al., 1984). Two main approaches to modeling non-aneurysmal SAH have been used: (1) direct injection of blood into the subarachnoid space, or (2) endovascular perforation of a cerebral vessel. While each of these models allows for the study of how extravascular blood within the subarachnoid space affects various outcomes after SAH, none addresses the specific consequences related to the formation and spontaneous rupture of an intracranial aneurysm, which may have its own independent additive or blood-dependent synergistic effect on SAH outcome.

### Direct injection of blood

**Figure 1:**
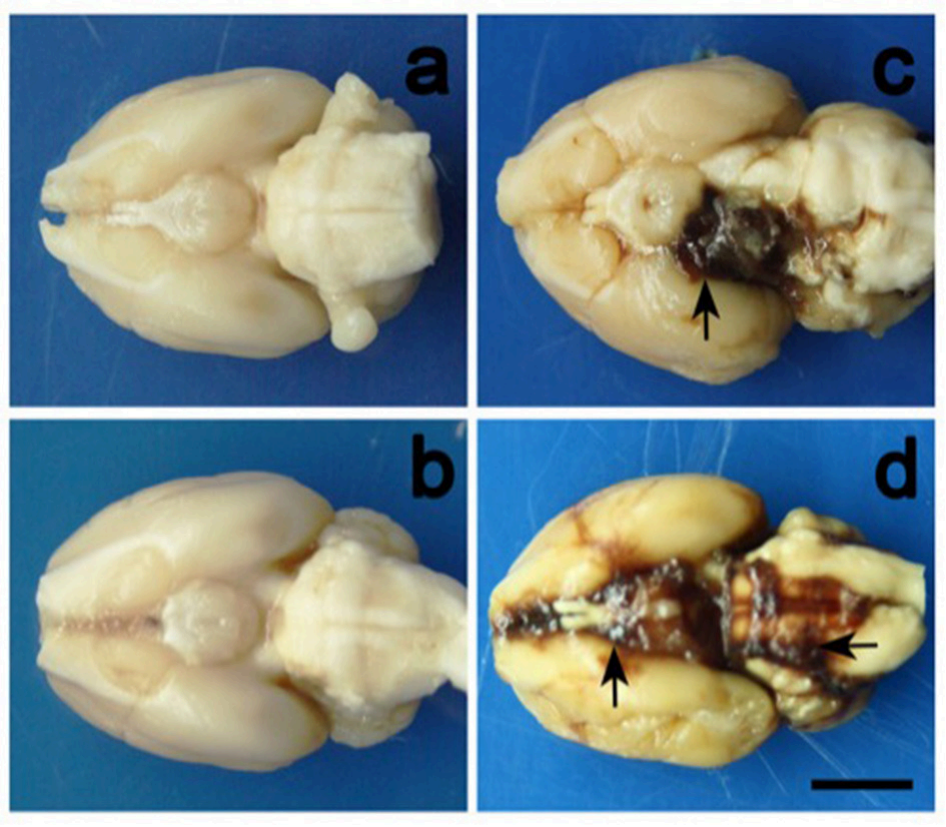
Representative illustrations of blood clot distribution in the cisterna magna and prechiasmatic cistern models. Images correspond to **(a)** cisterna magna control, **(b)** prechiasmatic cistern control, **(c)** cisterna magna experimental, and **(d)** prechiasmatic cistern experimental mouse brains. Cisterna magna and prechiasmatic cistern injections primarily result in blood clots surrounding the posterior and anterior circulations, respectively. The scale bar represents 2cm. Photo was obtained from Cai J. et al. (2012).

Direct injection of autologous or heterologous whole blood into the subarachnoid space is the most commonly used method of inducing non-aneurysmal SAH in rodents. During the procedure, stereotactic frames are used to produce precise coordinates for injection in an effort to control the location and distribution of blood in the subarachnoid space. Blood is either injected into the cisterna magna or prechiasmatic cistern, with each location producing a characteristic pattern of blood distribution. The former results in a blood clot primarily localized around vessels of the posterior circulation and the latter around vessels of the anterior circulation (Prunell et al., 2002; Raslan et al., 2012). Figure 1 provides a representative visualization of the blood distribution in the cisterna magna and prechiasmatic cistern injection models.

Direct injection models may involve a single or double injection of blood (Vatter et al., 2006; Weidauer et al., 2006; Lee et al., 2008, 2009; Güresir et al., 2010, 2012; Cai J. et al., 2012; Raslan et al., 2012; Boyko et al., 2013). In double injection models, the second infusion is typically performed 24 h after the first and injection occurs in the cisterna magna. Double injection of blood into the prechiasmatic cistern has not been performed, presumably because the hemorrhagic insult is more severe and the rodents may not be able to sustain two infusions. In general, less blood is required to produce the same deficits in the prechiasmatic cistern location compared to the cisterna magna site. The various blood volumes that have been introduced into both locations and the injection characteristics are summarized in Table 1.

Table 1Summary of single- and double-injection cisterna magna models and single-injection prechiasmatic cistern models in published studies that used various strains of mice or rats.

**Table:** 

**Rodent**	**Injection 1 (μL)**	**Injection length (s)**	**Injection rate (μL/s)**	**References**	**Comments**
**CISTERNA MAGNA—SINGLE INJECTION**					
C57BL/6 mice	60	60	1.0	Lin et al., 2003	Replaced 60 μL saline
SD rats	70 or 300	5 or 30	14 or 10	Delgado et al., 1985	
SD rats	70	–	–	Rasmussen et al., 1992	
SD rats	300	15	20.0	Boyko et al., 2013	
SD rats	300	15	20.0	Glenn et al., 2002	Replaced 300 μL saline
SD rats	300	–	–	Solomon et al., 1985	Replaced 300 μL donor blood
SD rats	300	15	20.0	Prunell et al., 2003	Attempted to keep ICP at the level of MABP
SD rats	300	30	10.0	Schwartz et al., 2000	
SD rats	300	120	2.5	Gules et al., 2002	Removed 300 μL CSF
SD rats	370	–	–	Solomon et al., 1987	Replaced 370 μL saline
SD rats	600	60	10.0	Swift and Solomon, 1988	
SD rats	500	1200	0.4	Ram et al., 1991	Removed 200 μL CSF
SD rats	300	180–240	–	Jackowski et al., 1990	Injections were made in 50 μL sequential steps, each over 3–4 min
Wistar rats	100	30	3.3	Munoz-Sanchez et al., 2012a	Removed 100 μL CSF
Wistar rats	200	–	–	Turowski et al., 2007	
C57BL/6 mice	60	–	–	Chaichana et al., 2007	Replaced 60 μL saline

**Table:** 

**Rodent**	**Injection 1 (μL)**	**Injection 2 (μL)**	**Injection length (s)**	**Injection rate (μL/s)**	**References**	**Comments**
**CISTERNA MAGNA—DOUBLE INJECTION**						
SD rats	200 or 300	100 or 200	600 or 180	0.33/0.17 and 1.67/1.11	Lee et al., 2008	
SD rats	200	100	60 and 30	3.3	Lee et al., 2009	
SD rats	200	200	180	1.1	Cai J. et al., 2012	Remove 100 μL CSF each time
SD rats	200	200	–	–	Vatter et al., 2006	Remove 100 μL CSF first time
SD rats	200	200	–	–	Weidauer et al., 2006	
SD rats	250	250	–	–	Güresir et al., 2010	Remove 100 μL CSF each time
SD rats	250	250	–	–	Güresir et al., 2012	Remove 100 μL CSF each time
SD rats	200	100	180	1.1 and 0.56	Raslan et al., 2012	Remove 100 μL CSF first time
SD rats	300	300	15	20.0	Boyko et al., 2013	
SD rats	300	300	120	2.5	Gules et al., 2002	48 h apart, remove 300 μL CSF each time
SD rats	300	300	120	2.5	Meguro et al., 2001	48 h apart, remove 100 μL CSF first time
Wistar rats	500	300	600	0.8 and 0.5	Takata et al., 2008	48 h apart, remove 200 μL CSF first time
SD rats	300	300	120	2.5	Wang et al., 2010	
SD rats	300	300	180	1.7	Qin et al., 2015	48 h apart, remove 300 μL CSF each time
SD rats	300	300	600	0.5	Zhang Z. Y. et al., 2015	48 h apart
SD rats	200	200	120	1.7	Zhao et al., 2016	48 h apart
SD rats	340–440	340–440	–	–	Chang et al., 2015a	48 h apart
SD rats	300	300	–	–	Chang et al., 2015b	48 h apart, removed 100 μL CSF each time
SD rats	200	100	80 and 40	2.5	He et al., 2015	48 h apart, removed 100 μL CSF each time

**Table:** 

**Rodent**	**Injection 1 (μL)**	**Injection length (s)**	**Injection rate (μL/s)**	**References**	**Comments**
**PRECHIASMATIC CISTERN—SINGLE INJECTION**					
CD1 mice	100	15	6.7	Sabri et al., 2009	
SD rats	200, 250, or 300	–	–	Prunell et al., 2002	Attempted to keep ICP at the level of MABP
SD rats	200	12	16.7	Prunell et al., 2003	Attempted to keep ICP at the level of MABP
SD rats	200	120	1.7	Cai J. et al., 2012	
SD rats	300	20	15.0	Jeon et al., 2010	
Wistar rats	300	60	5.0	Piepgras et al., 1995	
SD rats	300	180	1.7	Zhang D. et al., 2015	
SD rats	250	–	–	Ansar and Edvinsson, 2008	Injected at a pressure equal to that of MABP (80–100 mmHg)
SD rats	300	20	15.0	Zhang X. S. et al., 2015	

*Information presented includes rodent strain, injection volumes (μL), duration of injection (s), and injection rate (μL/s). Specific notes on the replacement volume of blood or saline, in addition to details regarding minor alterations to the model, are provided if mentioned in the referenced text. SD, Sprague Dawley*.

The direct injection model allows for a predictable distribution of blood, but can introduce variations in physiologic parameters. Given the large volume of blood and location of injection into the cisterna magna, pressure rises may cause blood to enter the spinal canal, potentially confounding results due to the functional impairments produced (Leonardo et al., 2012). In order to avoid this complication, many authors choose to remove CSF prior to injection to create more potential space for blood (Ram et al., 1991; Takanashi et al., 2001; Gules et al., 2002; Vatter et al., 2006; Takata et al., 2008; Güresir et al., 2010, 2012; Cai C. Y. et al., 2012; Muñoz-Sanchez et al., 2012b; Raslan et al., 2012). Unfortunately, this can alter the ICP, potentially affecting all observed outcomes. Another source of error is often seen in autologous blood injection models, as some have attempted to maintain normovolemia after blood withdrawal and SAH induction by replacing equivalent volumes of saline (Solomon et al., 1987; Glenn et al., 2002; Lin et al., 2003) or donor blood (Solomon et al., 1985) into the systemic circulation. However, because total blood volume decreases following SAH, this step may alter results by keeping MABP artificially high. Additionally, cisterna magna models commonly keep the animal tilted from 20 to 40° angle after injection to facilitate blood distribution into the anterior circulation (Gules et al., 2002; Lee et al., 2008). This manipulation likely disrupts intracranial pressure (ICP) and other important physiological parameters.

As noted in Table 1, the amount of time over which blood is injected also varies widely among different researchers. Ideally, blood injection would occur at a rate that maintains a pressure similar to MABP in order to mimic the true pressure seen in a spontaneous arterial bleed. In an effort to adhere to these conditions, Prunell et al. (2002) attempted to keep ICP at the same level as MABP during manual injection of blood, rather than choosing constant injection rates (Prunell et al., 2002, 2003). Additionally, Ram et al. (1991) did not allow ICP to rise to over 25 mmHg at any point during the injection (Ram et al., 1991). While these elegant procedures eliminate possible confounding variables, they are nevertheless technically strenuous and difficult to reproduce.

### Endovascular perforation

In addition to the direct injection of whole blood, SAH can be simulated by endovascular perforation (Table 2). This model involves advancing a suture into the ICA until it perforates a vessel within the Circle of Willis. Briefly, the method involves surgically exposing the bifurcation of the common carotid artery (CCA) into the ICA and external carotid artery (ECA). The suture is then threaded through the ECA into the ICA and advanced into the Circle of Willis at the branch point of the ICA into the ACA and MCA (Bederson et al., 1995).

**Table 2:** Summary of endovascular perforation models in published studies that used various strains of mice or rats.

**Rodent**	**Suture size**	**References**	**Comment**
**ENDOVASCULAR PERFORATION**			
C57BL/6 mice	5–0	Feiler et al., 2010	
C57BL/6 mice	5–0	Parra et al., 2002	ECA ligated, blunted suture tip
SD Rats	3–0 and 4–0	Schwartz et al., 2000	
SD Rats	3–0	Gules et al., 2002	
SD rats	3–0	Prunell et al., 2003	
SD rats	3–0, 4–0, or 5–0	Westermaier et al., 2009a	Suture left in ECA for 20–30 min before advancing through ICA
SD rats	3–0	Westermaier et al., 2009b	CCA clamped before placing suture in ECA, suture left in ECA for 20–30 min before advancing through ICA
SD rats	3–0	Bederson et al., 1998	ECA ligated
SD rats	3–0	Bederson et al., 1995	ECA ligated
SD rats	4–0	Sugawara et al., 2008	ECA ligated
SD rats	0.076 mm tungsten filament	Park et al., 2008	ECA ligated, hollow tubing
SD rats	3–0	Silasi and Colbourne, 2009	ECA ligated
Wistar rats	3–0	Van Den Bergh et al., 2005	ECA ligated, ICA and carotid communicis clamped before suture threaded into ECA
Wistar rats	3–0	Veelken et al., 1995	Blunted suture tip
Wistar rats	4–0	Tiebosch et al., 2013	
C57BL/6 mice	5–0	Egashira et al., 2015a	Blunted suture tip
C57BL/6 mice	5–0	Siler et al., 2015	
C57BL/6 mice	5–0	Vellimana et al., 2011	ECA ligated
SD Rats	4–0	Suzuki et al., 2010	
SD Rats	4–0	Topkoru et al., 2013	
C57BL/6 mice	5-0	Bühler et al., 2015	ECA ligated
C57BL/6 mice	5–0	Sheng et al., 2011	
SD Rats	3–0	Hockel et al., 2012	
SD Rats	4–0	He et al., 2012	
SD Rats	4–0	Li et al., 2012	
C57BL/6 mice	5–0	Egashira et al., 2015b	Blunted suture tip
SD Rats	4–0	Britz et al., 2007	
SD Rats	4–0	Hasegawa et al., 2011	
SD Rats	4–0	Duris et al., 2011	
SD Rats	3–0	Shishido et al., 2015	
SD Rats	4–0	Li et al., 2016	
SD Rats	3–0	Huang et al., 2015	
SD Rats	3–0, 0.08 mm tunsgten filament	Hollig et al., 2015	Hollow tubing
SD Rats	0.075 mm tungsten filament	Xu et al., 2015	Hollow tubing

*Information presented includes rodent strain and the size of the suture. Details regarding alterations to the model, such as the use of a blunted suture tip or ligation of the ECA, are also included if mentioned in the referenced text. SD, Sprague Dawley; ECA, external carotid artery; CCA, common carotid artery; ICA, internal carotid artery*.

Unlike most direct injection models, the bleed produced via endovascular perforation occurs at MABP. Furthermore, no needle is inserted through brain structures, greatly reducing the risk of intracerebral hemorrhage or confounding alterations in ICP. However, the volume of blood produced by this model depends on the size of suture used to perforate the artery, and even with the same size suture, the amount of blood is variable from rodent to rodent (Schwartz et al., 2000). Researchers have used a number of suture sizes to control the hemorrhage severity. While the sutures are usually sharpened, some have chosen a blunted tip in order to prevent endothelial damage when passing the suture through the ICA (Veelken et al., 1995; Parra et al., 2002; Lee et al., 2009). For further protection, Park et al. (2008) used hollow tubing and a tungsten filament rather than a suture to avoid injury to the vasculature before puncture (Park et al., 2008). With this model, it is not possible to control whether the suture perforates either the ACA or MCA specifically (Bederson et al., 1995). In some cases, the ICA can even be perforated (Bederson et al., 1995). Thus, in addition to the variation in hemorrhage volume, differences in puncture location between rodents in a given study may result in a non-uniform blood distribution.

Another complicating factor of the endovascular perforation model is the common practice of ligating the ECA into a stump to facilitate advancing the suture through the CCA and into the ICA (Bederson et al., 1995, 1998; Schwartz et al., 2000; Gules et al., 2002; Parra et al., 2002; Prunell et al., 2003; Van Den Bergh et al., 2005; Park et al., 2008; Sugawara et al., 2008; Lee et al., 2009; Silasi and Colbourne, 2009). As a result of this ligation, CBF is increased on the ipsilateral side, potentially exacerbating the severity of hemorrhage for a given filament size. In attempts to reduce extracranial blood loss as a result of ECA ligation, the CCA (Westermaier et al., 2009b) or ICA and carotid communicis (Van Den Bergh et al., 2005) have been clamped before placing the suture in the ECA. Finally, some researchers have left the suture in the ECA for 20–30 min before advancing it through the ICA, in order to obtain baseline measurements for data analysis (Schwartz et al., 2000; Westermaier et al., 2009a,b).

## Preclinical models of aneurysmal SAH

Thus far, the rodent models discussed do not involve aneurysm formation and rupture, even though non-aneurysmal SAH only represents ~10% of human SAH cases (Marder et al., 2014). The endothelial changes and local pro-inflammatory state associated with development of an aneurysm and its subsequent rupture may contribute to SAH outcomes. However, the incidence of spontaneous cerebral aneurysms in rodents is extremely low (Handa et al., 1983; Kim and Cervos-Navarro, 1991), making true aSAH difficult to study in rodents. Without such an understanding of these potential aneurysm effects, challenges arise in evaluating putative preventative and therapeutic paradigms in experimental models.

In an attempt to address the discrepancy between experimental models and clinical reality, extensive effort has been extended to the study of intracranial aneurysm induction in rodents. Methods involving hypertension and hemodynamic stress can result in aneurysm formation, although the aneurysms are relatively small and can take as long as 3 months to develop (Handa et al., 1983; Hashimoto et al., 1984; Li et al., 2014). Elastase can also be injected to degrade the internal and external elastic lamina of cerebral vessels, causing aneurysm formation in ~3 weeks (Nuki et al., 2009; Hoh et al., 2010; Tada et al., 2011, 2014; Ruzevick et al., 2013; Wada et al., 2013; Hosaka and Hoh, 2014; Starke et al., 2014a,b; Shimada et al., 2015). Using this hypertension, hemodynamic stress, and elastase triad, others have characterized the first mouse model that featured intracranial aneurysm formation (Nuki et al., 2009; Wada et al., 2013). In the method, C57BL/6J mice were injected with elastase at the right basal cistern and continuously infused with angiotensin-II to produce the desired hypertension and hemodynamic stress (Nuki et al., 2009). Accordingly, intracranial aneurysms of 500 μm size were produced, exhibiting a dose-dependent relationship between aneurysm incidence and concentrations of both elastase and angiotensin-II.

The choice of hypertensive agent is a key factor to consider in an aSAH model. Angiotensin-II can be used as the hypertensive agent (Nuki et al., 2009; Kanematsu et al., 2011; Pena Silva et al., 2014; Chu et al., 2015b), supported by data demonstrating that angiotensin-converting enzyme inhibitors can attenuate aneurysm rupture (Li et al., 2014). However, administering angiotensin II to promote aneurysm rupture may have confounding effects through its involvement in systemic inflammation and reactive oxygen species generation in the vessel wall (Tada et al., 2011, 2014). As an alternative, deoxycorticosterone acetate (DOCA) and saline can also induce intracranial aneurysm formation and rupture in a dose-dependent manner (Tada et al., 2014). Using a model involving unilateral nephrectomy, subcutaneous DOCA pellet implantation, 1% NaCl drinking water supplementation, and elastase injection, aSAH can successfully be induced in mice (Makino et al., 2012; Wada et al., 2013; Peña-Silva et al., 2015; Shimada et al., 2015). With this methodology, intracranial aneurysms form in the Circle of Willis and spontaneously rupture between days 7 and 16 after aneurysm induction, and rupture is reliably indicated by a simple assessment of neurological symptoms in the mice (Wada et al., 2014). While this novel approach offers promising results in developing an improved aSAH model in mice with features that are reflective of human parameters, limitations exist that can pose problems in experimental settings. First, the practice of unilateral nephrectomy can alter systemic levels of renin and other hormones affecting systemic blood pressure (Tada et al., 2014). In light of this, it is possible to achieve similar levels of systemic hypertension with the subcutaneous DOCA implants supplemented with 1% NaCl drinking water alone without altering the physiology of the anatomical organs in charge of the renin-angiotensin system (Klanke et al., 2008; Amann et al., 2009; Hartner et al., 2009; Rinne et al., 2013), although this has not been investigated in the setting of cerebral aneurysms. Furthermore, mice are typically euthanized to confirm aneurysm rupture after the onset of neurological symptoms, precluding further measurements of longitudinal aSAH outcomes unless *in vivo* imaging studies are performed. Additionally, while reflective of the unpredictable clinical course of aSAH, the spontaneous nature of aneurysm rupture in this model prevents synchronization of experimental rodent groups.

## Comparison of preclinical SAH models and clinical SAH

Direct injection models are widely employed because they allow investigators to control the initiation, volume, and rate of hemorrhage; although, the volumes used, and injection rates vary widely across studies. Additionally, the inability to simulate vessel rupture limits the translatability to clinical SAH. Alternatives such as the endovascular perforation model do incorporate vessel rupture, and this model is also advantageous in simulating several important physiologic parameters of clinical SAH, including direct entry of blood from the vasculature that occurs at MABP. However, there are several potential variables that can affect hemostasis and outcome in this model. Unfortunately, the amount and location of blood is not as controllable as with the direct injection methods and there is significant chance of artificial injury to the vasculature when advancing the suture. Finally, neither the injection nor endovascular perforation models include the formation and rupture of an aneurysm (Table 3). Further optimization of a spontaneous aneurysm formation and rupture model of aSAH is needed. If such a standardized and translational model can be developed, it would be optimal for studying the pathophysiology of aSAH and evaluating putative therapeutic avenues to improve outcomes.

**Table 3:** Presents a summary of the advantages and disadvantages of the two blood injection and endovascular perforation preclinical SAH models.

**Model**	**Advantages**	**Disadvantages**
Endovascular perforation	Bleed is at physiologic MABP	Inability to control the hemorrhage volume
	No needle insertion through brain structures	Artificial injury of vasculature
	Reduced risk of ICH	ECA ligation
Cisterna magna	Controlled hemorrhage volume	Lacks vessel rupture
		High variability in physiological parameters
		Head rotation artificially elevates ICP
Prechiasmatic cistern	Controlled hemorrhage volume	Lacks vessel rupture
	Reproducibility	
	Most translational with respect to outcomes	

## Cerebral vasospasm and delayed cerebral ischemia in rodent models of SAH

Following SAH, in addition to EBI, the most dreaded complication is cerebral vasospasm (CV), a prolonged narrowing of cerebral arteries resulting in diminished perfusion in the tissue distal to the narrowing (Greenberg et al., 2000). The consequences of CV include DCI, infarction, and diffuse edema, leading to poor outcomes for patients experiencing this unpredictable vascular event (Biller et al., 1988). In light of the delayed nature of CV and severe consequences, it is necessary to develop preventative measures and treatments for CV that can attenuate its ominous effects. In order to accomplish this task, efforts should be placed on developing a rodent model of aSAH that reproducibly yields delayed CV in a way that reflects the timing, location, and severity of clinical SAH.

### Identification of cerebral vasospasm in rodent models of SAH

Various methods have been developed and used to assess the occurrence and severity of CV following experimental SAH, each with their advantages and disadvantages. The most straightforward method of identifying CV is via histological analysis using fixed coronal brain slices and measuring the intraluminal or adventitial diameter of photomicrographs (Bederson et al., 1998; Meguro et al., 2001; Alkan et al., 2002; Gules et al., 2002; Lee et al., 2008, 2009; Park et al., 2008; Sugawara et al., 2008; Sabri et al., 2009; Güresir et al., 2010, 2012; Jeon et al., 2010; Cai J. et al., 2012; Raslan et al., 2012). Ideally, perfusion is performed with reagents at 37°C to avoid thermoregulatory vasoconstriction; although, most protocols either do not specify perfusion temperatures or document using ice-cold solvents (Lord et al., 2012). While this method proves experimentally convenient, varying degrees of dehydration among brain samples can result in significant differences in measured vessel diameters (Cai J. et al., 2012). Indeed, Cai and colleagues showed that the intraluminal diameter was much smaller in post-mortem histological analysis compared to synchrotron radiation angiography, an *in vivo* method (Cai J. et al., 2012). Furthermore, histological analysis is a terminal measurement, precluding the ability to repeat measurements of CV in the same animal at different time points.

In addition to histological analysis, some researchers identify CV via gel casting of the cerebral vasculature (Parra et al., 2002; Lin et al., 2003; Takata et al., 2008; Altay et al., 2009). Briefly, animals are perfused with 10% formalin, followed by perfusion with a combination of gelatin and India ink. Cerebral vessels are imaged using a video-linked dissecting microscope, and diameters are measured from the digitized images. While this method avoids desiccation seen in traditional histological analysis, limitations still exist. Parra and colleagues showed that the perfusion pressure of the gelatin cast expands the vessels, removing measurable CV when rats are perfused at pressures greater than MABP (Parra et al., 2002). Additionally, the group found that particulate and air emboli within the gelatin fixative could induce artifact that resembled CV histologically (Parra et al., 2002). In light of these findings, it is recommended that perfusion pressure remain at MABP to avoid increasing luminal diameter of vessels. While some studies document perfusing animals at pressures close to physiological values (Gules et al., 2002; Sugawara et al., 2008), many studies either do not report perfusion pressures or document values that tend to be higher than the MABP (Parra et al., 2002; Takata et al., 2008).

Many researchers have addressed the aforementioned issues by using angiography to study the rodent cerebral vessels (Delgado et al., 1985; Verlooy et al., 1991; Piepgras et al., 1995). Since rodent vessels are too small for accurate measurement with typical angiographic techniques, synchrotron radiation angiography and digital subtraction angiography are used to visualize vessel diameter *in vivo* (Vatter et al., 2006; Weidauer et al., 2006; Turowski et al., 2007; Cai J. et al., 2012). These methods employ radiologic techniques resulting in images with higher resolution. However, their use in assessing CV is limited due to the toxicity of the contrasts used. Indeed, angiography appears to remain a terminal measure that cannot be used to obtain serial *in vivo* measurements of CV.

A possible solution for measuring CV serially *in vivo* may be found in MRI. In 2005, Van Den Bergh and colleagues used MRA to determine the degree of CV in the rat, employing 3D time of flight images to measure vessel diameter (Van Den Bergh et al., 2005). The results produced were similar to those obtained in histological or angiographic methods (Bederson et al., 1998; Gules et al., 2002; Parra et al., 2002; Sugawara et al., 2008; Lee et al., 2009). Furthermore, the resolution achieved via MRI can be greater than angiographic methods, especially when 4.7T magnets are used. However, the method is disadvantageous in that MRI can be both time-consuming and expensive, and the rodent must be anesthetized for the procedure which itself could affect CV pathophysiology and the neuroinflammatory milieu following SAH.

### Arteries affected by cerebral vasospasm in rodent models of SAH

In the current rodent models of SAH, the location of CV appears to be dependent on the site of hemorrhage and the model used (Table 4). For example, in rat models employing a single injection of blood within the cisterna magna, CV predominantly occurs in the basilar artery (BA) (Delgado et al., 1985; Ram et al., 1991; Gules et al., 2002), and less frequently in the posterior communicating artery (Pcom) (Gules et al., 2002). In rat cisterna magna models employing double injection, CV also primarily occurred in the BA (Meguro et al., 2001; Vatter et al., 2006; Lee et al., 2008, 2009; Takata et al., 2008; Güresir et al., 2012; Raslan et al., 2012), and the Pcom (Meguro et al., 2001); however, CV was additionally observed in both the ACA (Lee et al., 2009; Cai J. et al., 2012) and MCA (Takata et al., 2008). This difference is likely due to the greater volumes of blood introduced into the subarachnoid space with repeated hemorrhage in the double injection models that allows for greater overall dispersal. Furthermore, when single injections were made in the rat prechiasmatic cistern rather than the cisterna magna, CV was predominantly found in the ACA (Jeon et al., 2010; Cai J. et al., 2012) and MCA (Piepgras et al., 1995; Sabri et al., 2009; Cai J. et al., 2012). In contrast to these rat studies, mouse single injection cisterna magna models elicited CV not only in the BA, but also in the ACA and MCA (Lin et al., 2003). This discrepancy is perhaps due to the smaller size of the mouse cranial vault compared to the rat and thus a larger clot distribution.

**Table 4:** Summary of cerebral vasospasm itemized by the model used for SAH induction in published studies that used various strains of mice or rats.

**Rodent**	**Location**	**Severity (% Reduction)**	**Time**	**References**	**Comments**
**CISTERNA MAGNA-SINGLE INJECTION**					
SD rats	BA	50	3 d	Ram et al., 1991	CV measured using photographs of the BA
SD rats	BA	34 or 40^*^ and 23 or 27^*^	10 min, 2 d	Delgado et al., 1985	Blood volumes of 70 or 300^*^μL
C57Bl/6 mice	BA	24, 11, 12, 13	6 h, 12 h, 24 h, 36 h	Lin et al., 2003	No CV at 1 h, 2 d, 3 d, 4 d, or 7 d
	ACA	27, 38, 24, 19, 16, 21	6 h, 12 h, 24 h, 36 h, 48 h, 72 h		No CV at 1 h, 4 d, or 7 d
	MCA	21, 16, 15, 12	6 h, 12 h, 24 h, 36 h		No CV at 1 h, 2 d, 3 d, 4 d, or 7 d
SD rats	BA	20	2 d	Gules et al., 2002	No CV at 7 d
	Pcom	20	2 d		No CV at 7 d
Wistar rats	ICA, ACA, MCA, and PCA	No significant difference	5 d	Turowski et al., 2007	
C57Bl/6 mice	BA	9	24 h	Chaichana et al., 2007	
C57Bl/6 mice	MCA	18	6 h	Luo et al., 2016	CV measured via two-photon imaging
SD rats	BA	20	5 d	Raslan et al., 2012	
**CISTERNA MAGNA-DOUBLE INJECTION**					
SD rats	BA	47	5 d	Vatter et al., 2006	No CV at 2 d, 3 d, 7 d, 9 d
SD rats	BA	9 and 46	3 d, 5 d	Güresir et al., 2012	
SD rats	BA	7 and 45	3 d, 5 d	Güresir et al., 2010	
SD rats	BA	40	5 d	Raslan et al., 2012	
SD rats	BA	36	5 d	Lee et al., 2008	No CV immediately after SAH
Wistar rats	BA	20	3 d	Takata et al., 2008	No CV at 7 d
	MCA	23	3 d		No CV at 7 d
SD rats	BA	32	5 d	Weidauer et al., 2006	No CV at 11 d
SD rats	BA	37 and 32	5 d, 7 d	Meguro et al., 2001	
	Pcom	39 and 46	5 d, 7 d		
SD rats	BA	4 and 10	90 min, 24 h	Lee et al., 2009	
	ACA	23 and 26	90 min, 24 h		
SD rats	ACA	51, 50, 44, 30	1 d, 3 d, 5 d, 7 d	Cai J. et al., 2012	
	MCA	59, 57, 52, 42	1 d, 3 d, 5 d, 7 d		
SD rats	BA	33	7 d	Gules et al., 2002	
	Pcom	35	7 d		
SD rats	BA	38	5 d	Wang et al., 2010	
SD rats	BA	40, 36, 22	3 d, 5 d, 7 d	Zhao et al., 2016	
SD rats	BA	50 and 46	3 d	Chang et al., 2015a	
SD rats	BA	47	3 d	Chang et al., 2015b	
SD rats	BA	67	3 d	He et al., 2015	
**PRECHIASMATIC CISTERN-SINGLE INJECTION**					
SD rats	ACA	55, 60, 32, 31	1 d, 3 d, 5 d, 7 d	Cai J. et al., 2012	
	MCA	56, 64, 50, 44	1 d, 3 d, 5 d, 7 d		
SD rats	ACA	50	8 d	Jeon et al., 2010	
CD1 mice	MCA	51	7 d	Sabri et al., 2009	
	ACA	No significant difference	7 d		
Wistar rats	MCA	16	2 d	Piepgras et al., 1995	
**ENDOVASCULAR PERFORATION**					
SD rats	ICA	17	1 d	Sugawara et al., 2008	
Wistar rats	ICA	12	2 d	Van Den Bergh et al., 2005	
C57Bl/6 mice	MCA	57	3 d	Parra et al., 2002	
SD rats	ICA	51	60 min	Bederson et al., 1998	
	ACA	43	60 min		
SD rats	ACA	45 and 48	90 min, 24 h	Lee et al., 2009	
	BA	33 and 44	90 min, 24 h		
SD rats	BA	17	2 d	Gules et al., 2002	No CV at 7 d
	Pcom	25	2 d		No CV at 7 d
C57Bl/6 mice	MCA	27	2 d	Vellimana et al., 2011	
C57Bl/6 mice	ACA	33	3 d	Sheng et al., 2011	
	MCA	29	3 d		
	ICA	31	3 d		
SD rats	BA	43	5 d	Qin et al., 2015	
	MCA	23	5 d		
	ACA	42	5 d		
SD rats	BA	33	3 d	Huang et al., 2015	

*Information presented includes rodent strain, severity, and location of cerebral vasospasm, and the timing of vasospasm post-SAH induction. Severity of cerebral vasospasm is measured as the percent reduction in vessel diameter. CV, cerebral vasospasm, SD, Sprague Dawley; BA, basilar artery; ACA, anterior cerebral artery; MCA, middle cerebral artery; Pcom, posterior communicating artery; PCA, posterior cerebral artery; ICA, internal carotid artery*.

In the endovascular perforation model, CV is observed in the ICA (Bederson et al., 1998; Parra et al., 2002; Van Den Bergh et al., 2005; Sugawara et al., 2008), ACA (Bederson et al., 1998; Lee et al., 2009), MCA (Parra et al., 2002), Pcom (Gules et al., 2002), and even the BA (Gules et al., 2002; Lee et al., 2009). This variability in the CV location is likely due to an inability to directly control both the specific hemorrhage location and the amount of blood in this model. In contrast, when Altay and colleagues specifically transected a vein in the cisterna magna of mice simulating non-aneurysmal SAH, they noted CV only in the MCA (Altay et al., 2009).

### Timing of cerebral vasospasm in rodent models of SAH

The need for a reliable tool to measure the rodent vasculature *in vivo* is necessary to properly quantify the temporal nature of CV in these preclinical models. Initiation of CV is difficult to determine, as rodents are typically sacrificed to measure vessel diameter directly, preventing temporal observation of vessel narrowing. However, using the methodologies herein described, several studies have recorded chronological findings of CV occurrence in both the direct injection and endovascular perforation models of SAH.

In rodent single injection cisterna magna models, CV is most common at 2 d (Delgado et al., 1985; Gules et al., 2002), but has also been shown at 10 min (Delgado et al., 1985), 6 h (Lin et al., 2003), 12 h (Lin et al., 2003), 36 h (Lin et al., 2003), and 3 d (Ram et al., 1991). In double injection cisterna magna models, CV is most reproducibly found at 3 d (Takata et al., 2008; Güresir et al., 2010, 2012) and 5 d (Meguro et al., 2001; Vatter et al., 2006; Weidauer et al., 2006; Lee et al., 2008; Güresir et al., 2010; Raslan et al., 2012). Moreover, maximal narrowing of vessels in this model has been reported at 7 d (Dombovy et al., 1998; Lee et al., 2009). Injection into the prechiasmatic cistern resulted in CV at 2 d (Piepgras et al., 1995), 3 d (Cai J. et al., 2012), 5 d (Cai J. et al., 2012), 7 d (Sabri et al., 2009; Cai J. et al., 2012), and 8 d (Jeon et al., 2010). Finally, in endovascular perforation models, CV is seen at 1 h (Bederson et al., 1998), 90 min (Lee et al., 2009), 1 d (Sugawara et al., 2008; Lee et al., 2009), 2 d (Gules et al., 2002; Van Den Bergh et al., 2005), and 3 d (Parra et al., 2002).

Of these models, in regards to the development of CV, the double injection model into the cisterna magna has classically been cited as the most similar to humans, due to the paralleled maximal narrowing of cerebral vessels at 7 d (Dombovy et al., 1998; Lee et al., 2009). However, some researchers have studied CV in this model on 7 and 9 d after SAH and were not able to reproduce the findings (Vatter et al., 2006; Takata et al., 2008). Similarly, injection in the prechiasmatic cistern produces CV at 7 and 9 d (Sabri et al., 2009; Jeon et al., 2010; Cai J. et al., 2012). It remains unclear what model most reproducibly replicates clinical SAH pathophysiology in regard to the development of CV.

### Severity of cerebral vasospasm in rodent models of SAH

In addition to the temporospatial nature of CV in rodent SAH models, the severity of CV can be assessed and is an important consideration. It is generally regarded as the degree of vessel constriction, either as a decrease in the luminal diameter or cross sectional area (Sobey and Faraci, 1998), although some studies have reported a decrease in vessel perimeter (Meguro et al., 2001). Notably, there is no standardized method for quantifying the severity of CV; however, it can be expressed as the percent reduction in vessel size regardless of the methodology employed, as reflected in Table 4. Due to the lack of standardization, the severity of rodent CV ranges widely, from as low as 10% in some studies to 64% in others depending on location of CV and on the method used to measure vessel size. In single injection cisterna magna models, the degree of constriction ranges from 20 to 40% (Delgado et al., 1985; Gules et al., 2002; Lin et al., 2003). In double injection cisterna magna models, CV tends to be more severe, with a constriction ranging from 20 to 64% (Meguro et al., 2001; Gules et al., 2002; Vatter et al., 2006; Weidauer et al., 2006; Lee et al., 2008, 2009; Takata et al., 2008; Güresir et al., 2010, 2012; Cai J. et al., 2012; Raslan et al., 2012). In prechiasmatic cistern single injection models, the degree of CV is reported as 17–62% (Piepgras et al., 1995; Sabri et al., 2009; Jeon et al., 2010; Cai J. et al., 2012). Finally, endovascular perforation models show a vessel reduction of 10–57% (Bederson et al., 1998; Gules et al., 2002; Parra et al., 2002; Van Den Bergh et al., 2005; Sugawara et al., 2008; Lee et al., 2009). The vast ranges recorded in these studies once again exemplify the need for both a standardized model of SAH induction and method for quantifying CV.

### Cerebral vasospasm-induced neuronal death in rodent models of SAH

Among histopathological outcomes observed and reported, neuronal cell loss is an important parameter for consideration in rodent SAH models. The causal mechanism of neuronal death after SAH can in part be attributed to EBI and to CV and subsequent ischemia. At 24 h, 5, 7 d, and as far as 8 d post-SAH, CV and neuronal death were observed simultaneously in rodent specimens (Lee et al., 2009; Sabri et al., 2009; Güresir et al., 2010; Jeon et al., 2010).

Several methods are available to evaluate neuronal death. Conventional techniques such as H&E staining (Prunell et al., 2003; Feiler et al., 2010; Güresir et al., 2010) can depict global necrosis of brain tissue and slightly more neuron-specific stains like cresyl violet can offer added specificity (Westermaier et al., 2009b). However, these stains detect features such as vacuolation and hyperchromatism that are not specific to neuronal degeneration, and are thus prone to false positives (Cammermeyer, 1961). Silver stains are more specific for degenerating neurons, but are more time-consuming and intensive (de Olmos et al., 1994). Addressing these issues, TUNEL and Fluoro-Jade have been used to assess tissues for degenerating neuronal cells (Takata et al., 2008; Lee et al., 2009; Sabri et al., 2009; Silasi and Colbourne, 2009; Jeon et al., 2010). TUNEL reveals DNA breaks in cells undergoing programmed cell death via an immunohistochemical staining procedure (Gavrieli et al., 1992), while Fluro-Jade detects the cell bodies, dendrites, axons, and axon terminals of degenerating neurons via an acidic fluorophore that binds specifically to dying neurons (Schmued et al., 1997). Fluoro-Jade can identify both apoptotic and necrotic cells, as opposed to the apoptosis-specific TUNEL method; as such, the neuronal damage assessed with TUNEL is often less pronounced than that identified with Fluoro-Jade (Lee et al., 2009). For example, apoptotic cells were not observed in the mouse hippocampus subjected to TUNEL in a 2009 study although neuronal injury was visualized in that region using Fluoro-Jade imaging (Sabri et al., 2009).

Regardless of the staining method used, neuronal damage is commonly observed in the hippocampus and cortex in all the rodent SAH models assessing this outcome (Prunell et al., 2003; Takata et al., 2008; Lee et al., 2009; Westermaier et al., 2009b; Feiler et al., 2010; Güresir et al., 2010; Jeon et al., 2010). Neuron death can also occur in regions such as the cerebellum (Jeon et al., 2010) and basal ganglia (Lee et al., 2009). Interestingly, the study identifying necrotic cells in the cerebellum utilized a prechiasmatic cistern injection model of SAH, thus observing neuronal damage in a location relatively distant from the injection site (Jeon et al., 2010). In contrast, cisterna magna injection models produce neuronal death in the hippocampus and cerebellum, locations in close proximity to the clot site (Takata et al., 2008; Lee et al., 2009; Güresir et al., 2010; Jeon et al., 2010). In a 2003 study comparing the incidence of neuronal cell loss between SAH models, only 11% of rats in the perforation model exhibited neuronal loss compared to 28 and 44% of rats in the cisterna magna and prechiasmatic cistern models, respectively (Prunell et al., 2003). Additionally, Lee et al. (2009) showed that neuronal degeneration has a tendency for sidedness in the endovascular perforation model, where cell death occurs more frequently ipsilateral to the puncture (Lee et al., 2009).

### Molecular pathways of cerebral vasospasm

Multiple molecular pathways of CV have been proposed, including nitric oxide scavenging, disruption of endothelin-1 (ET1), toxicity of blood breakdown products, and inflammation. It is likely that not one pathway is responsible for the development of CV, but rather that each of these pathways is acting concurrently and influencing each other throughout the course of CV pathophysiology.

ET1 is a soluble factor primarily produced by the vascular endothelium and is a canonical potent vasoconstrictor (Sumner et al., 1992; Schneider et al., 2007). ET1 binds the ETA and ETB receptors expressed by the vascular smooth muscle cells resulting in vasoconstriction via phospholipase C activation, inositol trisphosphate (IP_3_) production, and calcium mobilization (Schneider et al., 2007). Following SAH, ET1 is produced by activated mononuclear leukocytes in the CSF and is elevated acutely in patients that develop CV and neurological deterioration (Fassbender et al., 2000; Thampatty et al., 2011). Given the potent and prolonged effects of ET1, it remains a top contender in mediating the development of CV, and, therefore, also remains a therapeutic target (Penn et al., 2015). Although, a recent meta-analysis of the four clinical trials investigating the use of clazosentan, an endothelin receptor antagonist, showed that the drug does reduce the incidence of CV and DCI, but does not significantly improve neurologic outcomes (Shen et al., 2013).

A second main theory for the molecular pathways involved in the development of CV is regarding the presence of red blood cells, and their main cellular component, hemoglobin, in close proximity to the major cerebral vessels traversing through the CSF (MacDonald and Weir, 1991; Zhang et al., 2001; Asleh et al., 2003; Buehler et al., 2009). This correlation is further strengthened by the known association between the volume of blood in the subarachnoid space and the severity of angiographic vasospasm (Kolias et al., 2009) and a study involving monkeys where removal of the blood clot was shown to reverse angiographic vasospasm (Zhang et al., 2001). More specifically, CV has its onset around day 3 after aSAH, peaks on days 6–8, and usually lasts 2–3 weeks (Kolias et al., 2009). Phagocytosis and lysis of RBCs occurs by 16–32 h, peaks around day 7, and continues for days, with clumps of intact RBCs still enmeshed in the arachnoid for up to 35 days (MacDonald and Weir, 1991). Furthermore, it has been documented that changes in hemoglobin concentrations within the CSF tend to mirror the evolution of CV, though the mechanisms by which extracorpuscular hemoglobin causes delayed arterial narrowing are multiple and poorly understood (Dreier et al., 2002; Nishizawa and Laher, 2005; Pluta et al., 2009). Possibilities include neuronal apoptosis, scavenging or decreased production of the vasodilator nitric oxide, increased ET1 levels, direct oxidative stress on smooth muscle cells, ROS production and lipid peroxidation of cell membranes, modification of potassium and calcium channels, and differential up-regulation of genes (Pluta et al., 2009). In addition to hemoglobin itself, its breakdown products heme, iron, bilirubin, and bilirubin oxidation products have been implicated in initiating oxidative stress and a toxic neuroinflammatory cascade that contributes to the development of CV (MacDonald and Weir, 1991; Clark and Sharp, 2006). Improving the clearance of blood products from the brain remains a viable therapeutic target following SAH, as it would also inhibit nitric oxide scavenging and thereby shift the balance toward a more vasodilatory environment.

### Comparison of cerebral vasospasm and delayed cerebral ischemia in preclinical models and clinical SAH

It is difficult to assess whether similar arteries are affected in preclinical models and clinical SAH due to the limited and varied number of arteries assessed in preclinical studies when compared to clinical counterparts. In addition, whereas in clinical SAH the timing of CV usually occurs 6–8 days post-stroke, in preclinical models the timing varies dramatically both within and between models. This discrepancy is particularly noteworthy given the implications of CV in poor functional outcomes following clinical SAH. Moreover, barriers to the reproducibility of CV in preclinical models hinder efforts to studying the mechanisms that underlie its pathophysiological sequelae, as well as its severity. One other barrier to studying severity of CV is the histopathological methods used to measure vessel narrowing, which often tend to warp the shape or size of the vessels prior to analysis. Finally, despite the severe clinical consequences of DCI in clinical SAH, it is frequently not observed in preclinical models.

## Pathophysiology of rodent models of SAH

In addition to the anatomic changes that occur in cerebral vessels following SAH, several physiological parameters are substantially affected. CBF, ICP, MABP, CPP change throughout the course of SAH pathophysiology, and these variables are related by the following equation:

$$\begin{array}{l} CPP=MABP-ICP \end{array}$$

Following the bleed, there is an acute rise in ICP, which is mirrored by a compensatory rise in MABP in an attempt to maintain CPP. This results in a decrease in both CPP and CBF if the magnitude and rate of change in ICP is greater than that of MABP (Young and Bowling, 2012). With time, CBF typically recovers due to the reflex rise in the MABP, unless CPP has decreased dramatically, in which case autoregulation is impaired and global ischemia ensues (McMullan et al., 2010).

**Figure 2:**
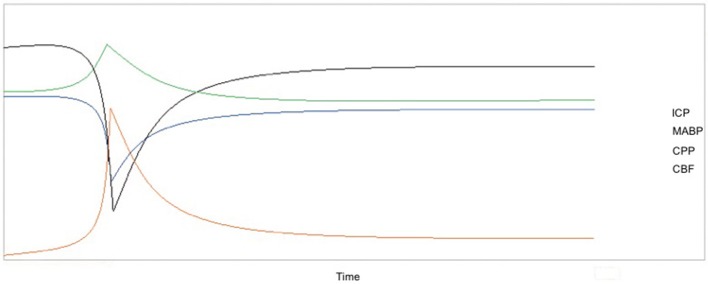
Temporal changes in CBF, ICP, MABP, and CPP over 1 h after induction of SAH with the x-axis representing the progression of time and the y-axis as relative change in each parameter. Due to the variation in the absolute values of these parameters after SAH (refer to Table 5), the relative changes in the variables are shown because these trends are preserved in nearly all preclinical studies and in humans. CBF and CPP sharply decrease shortly after SAH, followed by a return to near-baseline values within 1 h. Similarly, there is an acute increase in both ICP and MABP, followed by a return to baseline or near-baseline values within 1 h. Values for CBF were taken from studies that measured CBF using laser Doppler flowmetry.

These physiological parameters can be measured in preclinical SAH models. In non-aneurysmal SAH, all of these parameters follow the patterns seen in humans and return to near-baseline levels within 1 h post-induction of SAH. Figure 2 diagrams the interrelatedness between CBF, ICP, MABP, and CPP at baseline and within 1 h following experimental SAH in rodents. Additionally, Table 5 provides specific values for ICP, MABP, CPP, and CBF obtained from the various non-aneurysmal SAH models immediately following SAH and after 1 h (or as otherwise stated). These values have not been measured in aSAH mouse models due to the inability to predict the timing of spontaneous aneurysm rupture. As aSAH models are further developed and standardized, the quantification of physiological variables may be facilitated and improve our understanding of the differences in pathophysiology between the various SAH preclinical models and relevance to clinical SAH.

**Table 5:** Summary of pathophysiological outcomes itemized by the model used for SAH induction in published studies that used various strains of mice or rats.

**CISTERNA MAGNA-SINGLE INJECTION**														
**CBF**				**MABP**			**ICP**			**CPP**				
**Rodent**	**Acute (%Change)**	**Recovery (%Baseline)**	**Time**	**Acute (%Change)**	**Recovery (%Baseline)**	**Time**	**Acute (%Change)**	**Recovery (%Baseline)**	**Time**	**Acute (%Change)**	**Recovery (%Baseline)**	**Time**	**References**	**Comments**
SD rats	−42	Baseline	60 min	−10	Baseline	60 min	+463	234	60 min	−47	Baseline	60 min	Lee et al., 2009	
SD rats	−94/−94	55/50	60 min	+27	117	60 min	+1,625	18	60 min	–	–	–	Schwartz et al., 2000	Right hemisphere CBF/Left hemisphere CBF
SD rats	−63	Baseline	90 min	+5	Baseline	90 min	+1,300	243	90 min	−71	Baseline	90 min	Prunell et al., 2003	
SD rats	−43	57	60 min	Baseline	Baseline	60 min	+200	Baseline	60 min	–	–	–	Solomon et al., 1985	
SD rats	−50	57	3 h	+25	Baseline	3 h	+1,150	400	3 h	−20	85	3 h	Jackowski et al., 1990	
SD rats	−18	82	3 h	–	–	–	–	–	–	–	–	–	Swift and Solomon, 1988	
SD rats	−80	–	2 h	–	–	–	–	–	–	–	–	–	Raslan et al., 2012	
SD rats	–	–	–	+8	Baseline	1 d	–	–	–	–	–	–	Delgado et al., 1985	Preseted values pertain to animals injected with 300 μL of blood
SD rats	–	–	–	–	–	–	+1,212	400	60 min	–	–	–	Cai C. Y. et al., 2012	
C57Bl/6 mice	–	–	–	NS	Baseline	60 min	+270	292	30 min	–	–	–	Luo et al., 2016	
**CISTERNA MAGNA-DOUBLE INJECTION**														
SD rats	−48	75	60 min	−10	Baseline	60 min	+532	277	60 min	−75	87	60 min	Lee et al., 2009	
SD rats	−50	70	7 d	–	–	–	+1,212	400	60 min	–	–	–	Cai C. Y. et al., 2012	
SD rats	−32.5	110.5	1 d	–	–	–	–	–	–	–	–	–	Lee et al., 2008	
SD rats	−70	–	2 h	–	–	–	–	–	–	–	–	–	Raslan et al., 2012	
Wistar rats	–	73	14 d	–	–	–	–	–	–	–	–	–	Takata et al., 2008	
SD rats	–	–	–	NS	Baseline	–	–	–	–	–	–	–	Zhao et al., 2016	
**PRECHIASMATIC CISTERN-SINGLE INJECTION**														
Wistar rats	−47	80	30 min	−13^*^	Baseline	30 min	+662	190	30 min	−57	–	30 min	Piepgras et al., 1995	
SD rats	−69	89	90 min	+9	Baseline	90 min	+1,357	271	90 min	−77	Baseline	90 min	Prunell et al., 2003	
SD rats	−50	Baseline	7 d	–	–	–	+1,090	380	60 min	–	–	–	Cai J. et al., 2012	
SD rats	−94	Baseline	45 min	+27	Baseline	45 min	–	–	–	–	–	–	Jeon et al., 2010	
CD1 mice	−89	70	40 min	–	–	–	–	–	–	–	–	–	Sabri et al., 2009	
SD rats	–	–	–	+20	Baseline	60 min	+1,429	243	60 min	–	–	–	Prunell et al., 2002	
SD rats	−82	Baseline	1 h	–	–	–	+1,233	Baseline	60 min	–	–	–	Ansar and Edvinsson, 2008	
SD rats	–	–	–	NS	Baseline	–	–	–	–	–	–	–	He et al., 2015	
**ENDOVASCULAR PERFORATION**														
SD rats	−60	60	60 min	+40	89^*^	60 min	+1,175	339	60 min	−93	68	60 min	Lee et al., 2009	
C57Bl/6 mice	−81	80	30 min	+5	77	30 min	+1,439	588	30 min	−75	60	30 min	Feiler et al., 2010	
SD rats	−94^*^ and −94/−88 and −94	67^*^ and 75/70^*^ and 58	60 min	+29^*^ and Baseline	Baseline (both groups)	60 min	+1,522^*^ or +9,23	32^*^ or 20	60 min	–	–	–	Schwartz et al., 2000	Used filament sizes3–0^*^ and 4–0, respectively; Right hemisphere CBF/Left hemisphere CBF
SD rats	–	–	50 min	–	–	–	–	–	–	−67	75	50 min	Bederson et al., 1995	
SD rats	−83	44	60 min	–	–	–	+957	293	60 min	−66	83	60 min	Bederson et al., 1998	
SD rats	−29	Baseline	30 min	−20	Baseline	30 min	+332	–	30 min	–	–	–	Park et al., 2008	
SD rats	−78^*^ and −88	60^*^ and 45	6 h	+30	117	6 h	+817	367	6 h	−70	89	6 h	Westermaier et al., 2009b	Ipsilateral CBF^*^ and contralateral CBF, respectively
SD rats	−93, −81, and −68/−93, −81, and −74	47, 58, and 62/51, 57, and 78	120 min	+38, +38, and NS	Baseline, Baseline, and NS	120 min	+783, +433, and +233	467, Baseline, and Baseline	120 min	−46, NS, and NS	Baseline, NS, and NS	120 min	Westermaier et al., 2009a	Used filament sizes3–0, 4–0, and 5–0, respectively; Ipsi CBF/contra CBF
SD rats	−70	69	90 min	+25	Baseline	90 min	+1,243	414	90 min	−57	Baseline	90 min	Prunell et al., 2003	
C57Bl/6 mice	−45	81	30 min	15	Baseline	30 min	254	194	30 min	–	–	–	Siler et al., 2015	
C57Bl/6 mice	−88	75	45 min	–	–	–	1,060	340	45 min	–	–	–	Bühler et al., 2015	
SD rats	−75	74	60 min	–	–	–	1,400	400	60 min	−55	82	60 min	Hockel et al., 2012	
SD rats	–	–	–	NS	Baseline	10 min	948	377	10 min	–	–	–	Britz et al., 2007	
SD rats	−81	70	60 min	–	–	–	–	–	–	–	–	–	Ostrowski et al., 2006	
SD rats	–	–	–	–	–	–	755 and 580	–	90 min	–	–	–	Hollig et al., 2015	Classic technique and modified technique, respectively
SD rats	14	57	20 min	−26	Baseline	<5 min	–	–	–	–	–	–	Xu et al., 2015	
SD rats	−90	37	30 min	+16	Baseline	30 min	+564	231	30 min	55	Baseline	30 min	Huang et al., 2015	

*Particular focus is placed on key physiologic parameters that change after SAH, including CBF, CPP, ICP, and MABP. Information presented includes rodent strain, values for both the acute and recovery phases, and the post-SAH time at which the values were measured. Acute phase values are presented as the overall percent change, while the recovery phase values are presented as the percent of baseline. Missing values indicate that the study either did not measure that particular variable or that no value was mentioned in the paper. CBF, cerebral blood flow; MABP, mean arterial blood pressure; ICP, intracranial pressure; CPP, cerebral perfusion pressure; SD, Sprague Dawley*.

### Cerebral blood flow in rodent models of SAH

CBF is the most studied physiological variable in rodent models due to its importance in evaluating ischemia following SAH. Clinically, CBF exhibits a biphasic pattern: blood flow drops to a nadir near zero flow immediately after the bleed, followed by a return to levels slightly below baseline, decreasing once again if delayed CV occurs (Luft et al., 2004).

In order to evaluate CBF fluctuations following SAH in rodents, laser Doppler flowmetry (LDF) is most commonly used, although other methods such as MRI and autoradiography are also employed (Van Den Bergh et al., 2005; Tiebosch et al., 2013). LDF involves advancing a laser-emitting fiber optic probe into the epidural space of an anesthetized rodent and measuring changes in the wavelength of backscattered light detected by the probe as erythrocytes pass through vessels beneath it (Sutherland et al., 2014). This method obtains instantaneous measurements of relative changes in CBF and can be used at any time during SAH induction experiments. However, LDF is disadvantageous in that it measures cortical blood flow rather than total CBF. Additionally, it only provides temporal measurements, as any spatial information is limited by the location in which the probe is placed. Despite these limitations, LDF is the current method of choice in conducting rodent CBF measurements.

In the cisterna magna single injection models employing LDF, CBF drops acutely to 6–82% of baseline values, recovering to 58–100% of baseline values within 1 h (Schwartz et al., 2000; Prunell et al., 2003; Lee et al., 2009; Raslan et al., 2012). In the cisterna magna double injection model, there is an acute decrease to 30–52% of baseline after the first injection (Lee et al., 2009; Cai J. et al., 2012; Raslan et al., 2012); however, CBF tends to remain below baseline in these models, sometimes for as long as 2–3 d following injection (Lee et al., 2009; Cai J. et al., 2012). The return of CBF to original values following SAH thus depends on the number of injections and amount of blood injected into the cisterna magna. The notion that the double hemorrhage model imparts a greater physiologic insult than the single injection model is further evidenced by a study performed by Raslan and colleagues in which CBF fell to 20% below baseline initially and 30% below baseline at 5 d concurrent with CV in the BA (Raslan et al., 2012). In the prechiasmatic cistern injection model, the CBF nadir ranged from 6 to 31% of baseline with a return to ~80–100% of baseline values 1 h after SAH (Piepgras et al., 1995; Prunell et al., 2003; Sabri et al., 2009; Jeon et al., 2010; Cai J. et al., 2012). Finally, in the endovascular perforation model, the CBF nadir was between 6 and 71%, with regression to 44–81% of baseline values (Bederson et al., 1995, 1998; Schwartz et al., 2000; Prunell et al., 2003; Park et al., 2008; Lee et al., 2009; Westermaier et al., 2009a,b; Feiler et al., 2010). Based off of these values, the perforation model appears to be the most debilitating in terms of CBF, where the severity of the CBF reduction after perforation is likely due to the extent of insult, which is difficult to control with this method.

In addition to LDF, radiolabeled tracer molecules have also been used to measure CBF. This method involves injecting a chemically inert diffusible tracer such as [^14^C]*N*-isopropyl-*p-*iodoamphetamine into the circulation. Arterial blood is then withdrawn at a continuous rate, the animal is sacrificed, and brain tissue is extracted. A scintillation counter is used to measure the concentration of the tracer in both the arterial blood and brain sections, and CBF is calculated from these concentrations and the rate of blood withdrawal (Sakurada et al., 1978). Microspheres have been employed in a similar manner, but to date, both of these methods have been used only in single (Kim and Cervos-Navarro, 1991; Klanke et al., 2008) and double cisterna magna injection models (Delgado et al., 1985; Solomon et al., 1985; Swift and Solomon, 1988; Jackowski et al., 1990; Ram et al., 1991; Lee et al., 2008; Takata et al., 2008). Single injection models employing this technique show an acute decrease in CBF from 50 to 82% of baseline, with return to baseline values within a few days thereafter (Solomon et al., 1985; Swift and Solomon, 1988; Jackowski et al., 1990). One double injection model showed a 62% decrease in CBF, followed by a regression to initial values over 24 h; however, CBF then decreased once again to 70% of baseline at 5 d, again exhibiting a biphasic pattern (Lee et al., 2008). Overall, the recovery of CBF to near original values is highly variable, even when investigators inject similar volumes of blood in the cisterna magna: recovery was noted at 24 h (Swift and Solomon, 1988), 2 d (Jackowski et al., 1990), 7 d (Lee et al., 2008; Cai J. et al., 2012), and 35 d (Takata et al., 2008). The variations in observations, in conjunction with the terminal nature of experiments, make radiolabel tracer molecules less attractive than LDF or more recently developed radiographic techniques.

In light of the limitations of both LDF and radiolabeling methods, recent advances have been made in measuring rodent CBF using MRI (Van Den Bergh et al., 2005; Vatter et al., 2006; Güresir et al., 2010, 2012; Tiebosch et al., 2013). The method involves acquiring T1-weighted images that are ultimately constructed into perfusion maps used to calculate global CBF. Additional benefit is added in that these measurements of CBF can be conducted serially *in vivo*. In contrast to other studies up to that point, Van Den Bergh and colleagues found that there was no significant difference in CBF following SAH induced by endovascular perforation compared with injection models. Another endovascular perforation study demonstrated a baseline increase of 200% of CBF at 2 d and 150% at 7 d in both the ipsilateral and contralateral somatosensory cortex, contradictory to the findings of prior studies which document decreases in CBF (Tiebosch et al., 2013). Although justification is unclear for the discrepancy between MRI and LDF findings in perforation models, a possible explanation may be due to the fact that MRI measures global blood flow, while standard LDF only measures cortical flow in specific locations dependent upon probe placement. Furthermore, some areas of the brain may be hyperperfused in relation to others as a protective measure following SAH induction, which would not be identified using LDF alone. In addition to investigations with the perforation models, other studies have employed MRI to investigate the subacute stages of CBF following SAH in the cisterna magna injection models. In double hemorrhage models, CBF showed a 33–50% decrease at 3 d and 27–44% at 5 d (Vatter et al., 2006; Güresir et al., 2010, 2012). Not surprisingly, there was also marked CV at 5 d in each of these experiments, which was responsible for the delayed reduction in CBF (Vatter et al., 2006; Güresir et al., 2010, 2012). Unlike the recorded discrepancy in data for endovascular perforation models, the biphasic nature of CBF identified by MRI is in line with studies using LDF for the double injection models.

In conclusion, CBF can be measured in rodent models using LDF, radiolabeling methods, and MRI. Radiolabeling methods tend to have some variability and require euthanizing the animal to obtain the data output, making them less attractive. While LDF is easy to perform and is currently the mainstay of CBF measurements, its use is mainly limited to during SAH induction and immediately thereafter. On the other hand MRI is more time consuming, difficult to perform, and it is not plausible to measure CBF during the acute phase after SAH induction, as is commonly done with LDF. Although, MRI offers the benefit of serial measurements *in vivo* and the ability to measure global CBF and CBF in specific regions of interest such that correlations can possibly be made to the location of CV at that time.

### Intracranial pressure in rodent models of SAH

In addition to CBF, ICP is a commonly assessed physiological parameter following SAH. In the neurointensive care setting, increases in ICP are observed in over 50% of SAH patients (Badjatia et al., 2005). Typically, an ICP greater than 20 mmHg results in increased mortality and disability (MacDonald and Weir, 1991). In order to maintain an ICP within an appropriate range, the pressure is monitored continuously by insertion of a catheter through the parenchyma into the ventricles. The catheter is coupled to a pressure gauge that provides ICP values on a continuous basis (MacDonald and Weir, 1991).

Experimentally, a rise in ICP is often used as an indicator that SAH has occurred. As reflected in Figure 2, there is an acute rise in ICP after experimental SAH induction from the average rodent baseline of 5–7 mmHg, followed by a fall to either baseline or near baseline levels. While general trends in ICP can be outlined, measurements in rodent SAH models vary widely, perhaps due to the limitations of the method used to record ICP. At present, ICP is typically measured continuously via a catheter that is inserted into the rodent cranium through burr holes created in the calvarium, similar to the procedure done in humans.

More complications arise in injection models, as the added blood volume can alter ICP. Some studies attempt to correct for this confounding variable by not allowing ICP to increase above an arbitrarily-defined threshold while making the injection (Ram et al., 1991). Others have made multiple injections over a defined time period (Lacy and Earle, 1983), or attempted to keep the increase in ICP parallel to that of MABP during injection (Prunell et al., 2002, 2003). However, this is far from the ideal injection, which would occur at physiologic MABP. Furthermore, researchers often hold the rodent upside down following injection into the cisterna magna to facilitate blood distribution (Ram et al., 1991; Lin et al., 2003; Lee et al., 2008; Takata et al., 2008; Güresir et al., 2010, 2012; Cai J. et al., 2012; Munoz-Sanchez et al., 2012a). This step will falsely elevate the measured ICP. While injection models may elevate ICP erroneously due to the punctures created in the rodent cranium and subsequent maneuvers to distribute blood, these complications are not observed in the endovascular perforation model.

Despite the limitations in measuring ICP, important trends can be identified in the values obtained for this outcome following SAH. In cisterna magna single injection models, an acute rise from 18 mmHg to as much as 120 mmHg is observed following blood injection, with a subsequent decrease ranging from baseline values to 18 mmHg (Lacy and Earle, 1983; Solomon et al., 1985; Jackowski et al., 1990; Schwartz et al., 2000; Prunell et al., 2003; Lee et al., 2009; Cai J. et al., 2012). Interestingly, cisterna magna double injection models show a less dramatic increase, from 60 to 67 mmHg (Lee et al., 2009; Cai J. et al., 2012), followed by a reduction in ICP that remains consistently above baseline at 20–26 mmHg (Lee et al., 2009; Cai J. et al., 2012). In prechiasmatic cistern injections, the ICP rises to 46–107 mm Hg following injection, decreasing to values between 11 and 19 mmHg over time (Piepgras et al., 1995; Prunell et al., 2002, 2003; Jeon et al., 2010). Finally, in endovascular perforation models, the ICP acutely rises to values between 27 and 110 mmHg, subsequently decreasing to between 17 and 32 mmHg, which is higher than what is observed in injection models (Bederson et al., 1998; Schwartz et al., 2000; Prunell et al., 2003; Park et al., 2008; Lee et al., 2009; Westermaier et al., 2009a,b; Feiler et al., 2010).

### Cerebral perfusion pressure in rodent models of SAH

To date, direct measurement of CPP in rodents has not been described. Indirect quantification of CPP is possible via Equation (1). In this simple calculation, CPP is derived from the ICP and MABP, parameters that are easily measured using the methods outlined herein. Both MABP and ICP increase following SAH; however, the rise in MABP does not match that of ICP. Consequently, the CPP immediately following SAH falls. If the ICP rise is high enough, it may cause death due to lack of cerebral perfusion. Figure 2 depicts the pathophysiologic pattern of an immediate decrease in CPP after SAH, followed by a return to baseline levels within 1 h.

In cisterna magna single injection models, there is an acute 20–85% decrease in CPP, with recovery to baseline within about 1 h (Lacy and Earle, 1983; Jackowski et al., 1990; Prunell et al., 2003; Lee et al., 2009). The only study measuring CPP in a double injection model showed an acute decrease to 27% of baseline values, with a subsequent return to 92% of starting CPP (Lee et al., 2009). It is reasonable to suggest that the greater degree of hemorrhage in the double injection model prevents a return of CPP to initial values. Finally, in prechiasmatic cistern injection models, CPP acutely decreases to 23–49% of initial values, with recovery to baseline shortly thereafter (Piepgras et al., 1995; Prunell et al., 2003). In contrast to the injection models, more CPP data is available for studies inducing SAH using endovascular perforation. This model exhibits the greatest degree of CPP change; however, values are often inconsistent or contradictory. Acutely, there is a drastic decrease from 7 to 54% of initial CPP, followed by a return to 55–100% of baseline within an 1 h (Bederson et al., 1995, 1998; Prunell et al., 2003; Lee et al., 2009; Westermaier et al., 2009a,b; Feiler et al., 2010). One study even noted an extreme rise in ICP to 150 mmHg, resulting in a rapid drop of CPP and subsequent death of the rodents (Lee et al., 2009). Ultimately, the larger magnitude of CPP change observed in endovascular perforation models likely results from to the inability to control the degree of hemorrhage after filament insertion, in addition to the possibility of a longer duration of insult compared with injection models.

### Mean arterial blood pressure in rodent models of SAH

As reflected In Figure 2, MABP typically rises acutely following experimental SAH to preserve CPP and falls to baseline or near baseline levels thereafter, similar to the clinical counterpart.

The magnitude of the MABP increase observed in rodent studies varies with the SAH model and is typically measured by either a tail artery catheter, tail cuff sphygmomanometer (Sugawara et al., 2008; Zhao et al., 2011), femoral artery cannula (Lacy and Earle, 1983; Delgado et al., 1985; Jackowski et al., 1990; Schwartz et al., 2000; Prunell et al., 2003), or a radiotelemetry system (MacMillan et al., 2002; Pemberton et al., 2002; Zoerle et al., 2015). In 1992, Rasmussen et al. showed that the autoregulation of MABP and CBF was markedly disturbed as far as 5 d beyond induction of SAH in a single injection cisterna magna model, hypothesizing that such prolonged disturbance could possibly be due to delayed CV (Rasmussen et al., 1992). In additional single injection cisterna magna studies, the MABP acutely rose to 105–150% of baseline value immediately after SAH (Lacy and Earle, 1983; Delgado et al., 1985; Jackowski et al., 1990; Schwartz et al., 2000; Prunell et al., 2003). Regardless of this instance, the MABP values for single injection cisterna magna models predominantly returned to baseline values over time (Delgado et al., 1985; Jackowski et al., 1990; Prunell et al., 2003; Lee et al., 2009). In double injection cisterna magna models, only one study recorded MABP data, showing an acute 90% decrease, followed by a return to initial values, further demonstrating that autoregulatory mechanisms may be disrupted following SAH induction (Lee et al., 2009). In prechiasmatic cistern injection models, MABP rises to 109–127% of baseline values, followed by recovery to initial MABP (Prunell et al., 2002, 2003; Jeon et al., 2010; Cai J. et al., 2012).

Endovascular perforation models generally produce higher transient MABPs than injection models. However, these results vary greatly depending on the size of the suture employed (Prunell et al., 2003; Lee et al., 2009). For example, perforation with a 4-0 prolene suture will not significantly raise the MABP, while a 3-0 suture will produce a MABP higher than that induced by a 300 μL autologous blood injection in the cisterna magna (Schwartz et al., 2000). Furthermore, one study recorded a transient drop in blood pressure after perforation rather than the expected rise (Park et al., 2008). As in the case of the similar prechiasmatic cistern study, this drop may be attributed to a failure in autoregulatory mechanisms due to the hemorrhagic insult. Overall, following SAH induced by endovascular perforation, MABP will typically increase to 105–140% of baseline values (Schwartz et al., 2000; Prunell et al., 2003; Park et al., 2008; Lee et al., 2009; Westermaier et al., 2009a,b; Feiler et al., 2010). Unlike other models, the recovery is not always to initial values, but between 89 and 117% of MABP recorded prior to SAH induction (Schwartz et al., 2000; Prunell et al., 2003; Park et al., 2008; Lee et al., 2009; Westermaier et al., 2009a,b; Feiler et al., 2010).

### Comparison of pathophysiology in preclinical models and clinical SAH

Previous studies that utilized preclinical models of SAH have shown large variability in the absolute values of physiological parameters following induction of SAH both across and within different models. Whereas in clinical SAH the absolute value of these parameters typically depends on the magnitude of the hemorrhage, in preclinical models it can be influenced by a number of different factors, such as the model and surgical procedures used, anesthetics used, and measurement methods, particularly in the endovascular perforation model. On the other hand, trends in the relative change in these physiological parameters after the initial insult generally tend to be preserved between preclinical models of SAH and clinical SAH. In general, these variables can be assessed in rodent models of SAH, and new techniques are emerging to allow for more accurate measurement of such parameters. In optimizing these protocols, experimental methods should be standardized such that the data obtained in experimental models can not only be linked to functional outcomes in rodents, but can also be reliably correlated with the clinical picture of SAH in humans.

## Mortality and functional outcomes in rodent models of SAH

### Assessment of neurological function in rodent models of SAH

Studies of experimental SAH in rodents frequently include documentation of changes in rodent body weight. A reduction in weight after SAH tends to correlate with an overall decrease in neurological function. Overall, there is no apparent difference in magnitude of weight loss among the SAH models. In rats, weight loss following surgery ranged from 5 to 12%, regardless of which method was used to induce SAH (Delgado et al., 1985; Rasmussen et al., 1992; Glenn et al., 2002; Parra et al., 2002; Prunell et al., 2002; Kojima et al., 2005; Takata et al., 2008; Lee et al., 2009; Jeon et al., 2010). Uniquely, one endovascular perforation study conducted in the mouse showed a weight loss of ~20%, slightly higher than that seen in the rat models (Feiler et al., 2010).

Motor ability is another functional parameter observed after SAH, whether assessed with specific tests or with general observations of animal motility. The majority of studies showed that rodents are drowsy after surgery, but very few experience focal deficits or paralysis for up to 15 d past surgery (Barry et al., 1979; Delgado et al., 1985; Solomon et al., 1987; Swift and Solomon, 1988; Rasmussen et al., 1992; Bederson et al., 1995; Piepgras et al., 1995; Gules et al., 2002; Lin et al., 2003; Altay et al., 2009; Lee et al., 2009; Raslan et al., 2012). While, other investigations have reported rodent paresis following SAH (Prunell et al., 2003; Kojima et al., 2005; Lee et al., 2008; Raslan et al., 2012). For example, a study of the three major SAH models showed hemiparesis in 33% of rodents injected in the prechiasmatic cistern, compared with 14% in the single injection cisterna magna model and 11% in the perforation model (Prunell et al., 2003). Additionally, Kojimia et al. observed a light paresis in 36% of rodents subjected to endovascular perforation, attributing it to cerebral ischemia following SAH (Kojima et al., 2005).

Motor and behavioral function can be compositely evaluated with scoring systems analogous to those used clinically. A test developed by Bederson et al. (1986) observes forelimb flexion, resistance to lateral pushing, and circling behavior of rodents. Animals that have experienced ischemic events will incur a higher score on the Bederson scale (Rademaker et al., 2002b). Many protocols modify the scale to easily and serially detect neurological impairments after SAH (Parra et al., 2002; Vatter et al., 2006; Feiler et al., 2010; Güresir et al., 2010, 2012); however, the scales are limited due to the subjectivity in assessing each mouse, introducing variation in scores from observer to observer (Rosengart et al., 2007). Because of this setback, study results can be contradictory: some show the greatest decline in scores between 0 and 3 d following SAH with subsequent improvement (Parra et al., 2002; Vatter et al., 2006; Feiler et al., 2010), while others observe the most severe deficits at 5 d after SAH (Güresir et al., 2010, 2012). The worst neurological scores incurred on the Bederson scale occur between 0 and 5 d and are correlated with maximum CV (Vatter et al., 2006). Another scoring system is described by Garcia et al. which assesses motor activity through observations of spontaneous activity, symmetry of movement in the extremities, forepaw outstretching, climbing, lateral push, and vibrissae touch response (Garcia et al., 1995). Perforation models show a deficit by these criteria 1 d after SAH, which recovered thereafter, reaching near baseline levels at 7 d (Sugawara et al., 2008; Tiebosch et al., 2013). Using this scale, Cai et al. compared the prechiasmatic cistern injection model to the cisterna magna model, where the former had lower neurological scores at 3 d, although there was no significant difference between the groups (Cai J. et al., 2012). Additionally, a similar scoring system described by Feldmen et al. was used to compare a double to single hemorrhage cisterna magna model at 1 and 2 d, and weeks 1, 2, and 3 after SAH (Feldman et al., 1996; Boyko et al., 2013). Similar deficits resulted between both hemorrhage groups at 24 h after each injection, but deficits were attenuated by 1 week (Boyko et al., 2013).

Other tests include the use of apparatuses like grids, cylinders, ledge-tapered balance beams, pellet retrieval reaching chambers, staircases and ladder rungs to assess motor function (Rosengart et al., 2007). Sensorimotor function can be tested on apparatuses like the accelerated rotarod (Dunham and Miya, 1957). The use of these tests involves pre-training rodents for specific tasks before induction of SAH, followed by serial testing each day after hemorrhage (Takata et al., 2008; Silasi and Colbourne, 2009). Some studies have revealed an immediate decrease in function following SAH, with progressive improvement over a period of 4 weeks (Takata et al., 2008). In contrast, others observe no significant differences in skills with measurements conducted until 3 weeks following SAH (Silasi and Colbourne, 2009).

In addition to motor and sensorimotor assessment, cognitive impairments in spatial and working memory can be evaluated using the Morris Water Maze (MWM) task (Morris, 1984). The MWM involves placing animals in a round pool filled with opaque water and observing the rodents as they swim to a submerged platform to escape the water. Parameters such as initial heading angle, escape latency, swim time, and path length are measured and correlate to spatial learning and working memory functions. Trials with the MWM show increases in both swim time and distance between 3 and 5 weeks after SAH (Takata et al., 2008; Silasi and Colbourne, 2009). Another study showed increased escape latency at 2–5 d after hemorrhage; however, the increase in escape time was attributed to subacute motor deficits rather than memory deficits (Jeon et al., 2010). The same study showed no other deficits in working or reference memory on days 6–8, postulating that the neurologic deficits take more time to manifest (Jeon et al., 2010).

In addition to the cognitive function assessments, affective behavior tests include forced swim, elevated plus maze, sucrose preference, and open field tests (Boyko et al., 2013). Notably, rats that had undergone a double hemorrhage had worse deficits compared to single hemorrhage rats (Boyko et al., 2013).

### Cerebral vasospasm and delayed neurological deficits in rodent models of SAH

To accurately depict the clinical course of SAH in humans, delayed CV in rodents should result in neurological deficits due to cerebral ischemia. For example, Parra and colleagues showed a correlation between proximal MCA diameter and neurological score in a perforation model, indicating that CV played a role in neurological deficits following SAH in this model (Parra et al., 2002). Additionally, a cisterna magna model showed the worst neurological deficit at 5 d concurrent with the maximal degree of CV (Güresir et al., 2010, 2012). In a comparison of injection models, there was delayed CV at 3 d and 7 d with marked neurological deficits also appearing at 3 d (Cai J. et al., 2012). Departing from these trends, some injection models showed the greatest CV at 5 d, but yielded the worst neurological deficit at 0–3 d with attenuation by 5 d (Vatter et al., 2006; Lee et al., 2008). In the same manner, a prechiasmatic cistern model showed CV at 8 d, but no change in a working memory task completed that same day (Jeon et al., 2010). Although this case did not exhibit working memory defects, a spatial learning deficit manifested at 5 d, the final day of serial measurements of this parameter (Jeon et al., 2010). CV was not assessed at 5 d, inhibiting the correlation between spatial learning deficits and documented arterial narrowing.

In addition to these acute and subacute correlations, future studies are necessary that observe the effect of CV on long-term outcomes after SAH in rodents. Working and spatial memory tasks carried out weeks after hemorrhage result in significant neurological deficits (Takata et al., 2008; Silasi and Colbourne, 2009; Boyko et al., 2013), despite the fact that CV is largely attenuated in rodents roughly 1 week following SAH. These observations could be a result of neuronal death induced by DCI following CV, but additional studies are necessary to establish this correlation. Although studies have evaluated acute brain damage following hemorrhage with MRI (Van Den Bergh et al., 2005; Vatter et al., 2006; Güresir et al., 2010, 2012; Tiebosch et al., 2013), the damage has not been assessed during the weeks to months after the initial bleed. Further studies are necessary to evaluate the extent of neuronal damage in the rodent brain at these time points and its relation to longitudinal neurological function and overall survival.

### Mortality in rodent models of SAH

Rodent models of SAH exhibit a wide range of mortality (Table 6). Cisterna magna single injection models produce 0–16% mortality (Delgado et al., 1985; Solomon et al., 1985; Ram et al., 1991; Glenn et al., 2002; Gules et al., 2002; Lin et al., 2003; Prunell et al., 2003; Turowski et al., 2007; Munoz-Sanchez et al., 2012a; Boyko et al., 2013), while cisterna magna double injection models exhibit a greater range of 0–43% rate within the first few days (Glenn et al., 2002; Gules et al., 2002; Weidauer et al., 2006; Lee et al., 2008, 2009; Cai J. et al., 2012). At a later time point of 9 d following hemorrhage, mortality was as high as 53% in one double injection model (Vatter et al., 2006). The discrepancy in ranges between the two models is likely due to the amount of blood injected, with more blood increasing mortality risk. The prechiasmatic cistern injection model shows a higher mortality than the cisterna magna models, likely due to the hemorrhage location and smaller volume of the prechiasmatic cistern. In this model, mortality ranges from 10 to 33% within several days following hemorrhage (Prunell et al., 2002, 2003; Sabri et al., 2009; Jeon et al., 2010; Cai J. et al., 2012). However, mortality varies depending on the volume of blood administered. For example, Prunell and colleagues showed that a volume of 300 μL of autologous blood produces almost 100% mortality in rats 1 week post-injection, while 200 μL produces only 25% mortality (Prunell et al., 2002). Compared with injection models, endovascular perforation yields a higher mortality, with rates ranging from 16 to 66% (Bederson et al., 1995, 1998; Gules et al., 2002; Prunell et al., 2003; Kojima et al., 2005; Van Den Bergh et al., 2005; Park et al., 2008; Sugawara et al., 2008; Lee et al., 2009; Silasi and Colbourne, 2009; Feiler et al., 2010; Tiebosch et al., 2013).

**Table 6:** Summary of mortality itemized by the model used for SAH induction in published studies that used various strains of mice or rats.

**Rodent**	**Number**	**Mortality**	**References**	**Comment**
**CISTERNA MAGNA-SINGLE INJECTION**				
Wistar rats	13	23	Munoz-Sanchez et al., 2012a	3 SAH rats died during procedure, 1 within 48 h; 1 sham rat died
Wistar rats	7	0	Turowski et al., 2007	1 rat died due to anesthesia
SD rats	22	0	Glenn et al., 2002	1 rat died due to cannulation complications
SD rats	25	0	Ram et al., 1991	Some rats died during photography of the BA
SD rats	52	12	Delgado et al., 1985	0.3 mL blood injection + angiography at 5, 10, 15, 30, 60, 90 min and 1, 2, 3, 5, 7 d post-surgery; deaths attributed to respiratory failure secondary to obstruction of the tracheal tube
	6	0		0.07 mL injection of blood + angiography at 10 min and 2 d post-surgery
	8	0		0.3 mL injection of blood, no angiography
SD rats	6	0	Solomon et al., 1985	
C57Bl/6 mice	59	2	Lin et al., 2003	1 mouse died of respiratory failure immediately following SAH, 1 died of internal bleeding from an IP injection
SD rats	7	0	Prunell et al., 2003	
SD rats	10	0	Gules et al., 2002	
**CISTERNA MAGNA-DOUBLE INJECTION**				
SD rats	8 and 14^*^	25 and 43^*^	Güresir et al., 2010	3 d sacrifice; 5 d sacrifice^*^
SD rats	15 or 54^*^	40 or 2^*^	Lee et al., 2008	0.3/0.2 mL of blood injection; 0.2/0.1 mL blood injection^*^
SD rats	45	47	Vatter et al., 2006	6 rats died within 6 h of second blood injection, 5 rats between 2 and 3 d, 9 rats between 3 and 5 d, 1 rat at 6 d
SD rats	57	23	Cai J. et al., 2012	Mortality defined as death within 48 h of surgery
SD rats	23	0	Lee et al., 2009	
SD rats	11	9	Gules et al., 2002	Death occurred on the 3rd day after the second injection of blood
SD rats	19	21	Wang et al., 2010	Mortality defined as death within 120 h of surgery
SD rats	30	20	Zhang D. et al., 2015	Mortality defined as death within 48 h of surgery
SD rats	48	0	Zhao et al., 2016	
SD rats	25	20	Zhang D. et al., 2015	Mortality defined as death within 72 h of surgery
**PRECHIASMATIC CISTERN-SINGLE INJECTION**				
SD rats	54	13	Cai J. et al., 2012	Mortality defined as death within 48 h of surgery
SD rats	13	31	Jeon et al., 2010	Death occurred within 24 h of surgery
CD1 mice	10	10	Sabri et al., 2009	Death occurred within 24 h of surgery
SD rats	12	25	Prunell et al., 2003	
SD rats	4, 4^*^, 12^#^	100, 50^*^, 25^#^	Prunell et al., 2002	Groups injected with 0.3 mL, 0.25 mL^*^, and 0.2 mL^#^ autologous blood
SD rats	194	19	Zhang D. et al., 2015	
SD rats	–	4	Ansar and Edvinsson, 2008	Did not report sample size
SD rats	44	18	Zhang X. S. et al., 2015	
**ENDOVASCULAR PERFORATION**				
SD rats	25	57	Gules et al., 2002	4 animals died within 6 h, 6 within 6–24 h, and 3 within 24-48 h
SD rats	41	44	Lee et al., 2009	Death occurred within 24 h of arterial puncture; 7 rats died due to cardiac arrest following hemorrhage, the rest generally within 6 h
SD rats	45	20	Park et al., 2008	Mortality defined as death within 48 h of surgery
	95	46		Mortality defined as death within 48 h of surgery
SD rats	32	16	Sugawara et al., 2008	Stratifying mortality into the degree of hemorrhage gives a mortality of 0, 11, and 23.5 for rats that had mild, moderate, and severe SAH, respectively.
SD rats	21	57	Bederson et al., 1998	Mortality defined as death within 24 h of surgery at sacrifice point
	23	65		Animals sacrificed 60 min after surgery
	11	45		
Wistar rats	30	43 and 57	Tiebosch et al., 2013	Mortality defined as death within 48 h of surgery
SD rats	46	33	Silasi and Colbourne, 2009	All but two rats died within 12 h of surgery
SD rats	16	44	Prunell et al., 2003	86 of these rats died within 24 h
C57Bl/6 mice	10	30	Feiler et al., 2010	1 mouse died at 1 d, 2 mice died at 2 d
SD rats	16	50	Bederson et al., 1995	Death occurred within 24 h of surgery
Wistar rats	14	21	Kojima et al., 2005	Death occurred within 12 h of surgery
C57Bl/6 mice	58	19	Egashira et al., 2015a	Death occurred within 24 h of surgery
SD rats	12	17	Suzuki et al., 2010	Mortality defined as death within 24 h of surgery
	12	25		Mortality defined as death within 48 h of surgery
	12	17		Mortality defined as death within 120 h of surgery
	33	24		Mortality defined as death within 24 h of surgery
SD rats	35	34	Topkoru et al., 2013	Death occurred within 72 h of surgery
C57Bl/6 mice	10	10	Bühler et al., 2015	Death occurred within 7 d of surgery
C57Bl/6 mice	23	9	Sheng et al., 2011	Death occurred within 72 h of surgery
SD rats	10	50	Hockel et al., 2012	Death occurred within 72 h of surgery
SD rats	50	18	He et al., 2012	Did not specify the time points used for calculating mortality
SD rats	39	38	Li et al., 2012	Did not specify the time points used for calculating mortality
C57Bl/6 mice	17	24	Egashira et al., 2015b	Death occurred within 24 h of surgery
	10	20		Death occurred within 8 d of surgery
SD rats	39	15	Hasegawa et al., 2011	Mortality defined as death within 72 h of surgery
SD rats	34	29	Duris et al., 2011	Mortality defined as death within 6 h of surgery
SD rats	27	7	Ostrowski et al., 2006	Mortality defined as death within 6 h of surgery
SD rats	116	25	Shishido et al., 2015	Mortality defined as death within 24 h of surgery
SD rats	15 and 15	40 and 20	Hollig et al., 2015	Mortality defined as death within 24 h of surgery
SD rats	9	33	Xu et al., 2015	Death occurred within 24 h of surgery
SD rats	122	42	Huang et al., 2015	Did not specify the time points used for calculating mortality

*Information presented includes rodent strain, number of animals that died, and the percent of animals that died. Notes are included if the study mentions that animals died during a study procedure, if the investigators used a different definition for mortality, or if animals died within a short time period after the surgical procedure. SD, Sprague Dawley; BA, basilar artery*.

### Comparison of functional outcomes and mortality in preclinical models and clinical SAH

Similar to the physiological parameters previously discussed, functional outcomes and mortality in preclinical models of SAH appear to be highly variable within and across models. This is due in part to subjective nature of the tests and lack of inter-observer reliability, varied methods used to induce experimental SAH, varied mode of determining functional outcomes and time point used to assess mortality. It is clear that no studies exist to determine functional outcomes and mortality at long-term time points, something that is needed to assess the worthiness of the existing preclinical models in replicating the human disease process. Commonly, functional outcomes and mortality following clinical SAH is related to specific pathological factors, such as the extent of insult, and the occurrence of EBI, CV, and DCI, and baseline function must be taken into account. Although assessments of post-SAH functional outcomes have their limitations, the lack of focal deficits in the majority of preclinical studies does replicate the clinical counterpart. Finally, the biggest downfalls of preclinical models is their lack of replication of delayed neurological deficits coinciding with the onset of DCI, which are major contributors to poor outcomes following clinical SAH.

## Conclusions

Many rodent models of SAH and variations thereof exist in current preclinical experimentation. The most commonly used are the prechiasmatic and cisterna magna blood injection models and the endovascular perforation model. Each protocol offers its own advantages and disadvantages in observing outcomes of EBI, CV, physiologic parameters like CBF, and neurological deficits. However, all these models lack the spontaneous rupture of an intracranial aneurysm, which is needed for better replication of the early and delayed clinical aSAH pathophysiology. Furthermore, the lack of standardized procedures in the currently available models has led to considerable variation in the reporting of important outcomes, such as the onset, location, severity, and time course of CV making it difficult to compare across studies and to translate findings to clinical practice. Nonetheless, the prechiasmatic cistern anterior circulation model has been proposed as the most translational model in terms of reproducibility and outcomes (Attia and Loch MacDonald, 2015). With further optimization and continued research, a rodent model that parallels human SAH can be established. If this is achieved, translational capacity of preclinical models can be maximized and clinical interventions will improve as a result.

## Author contributions

All contributed to the design of the paper, participated in the search, compiled the tables, designed the figure, drafted, and reviewed the final version of the paper.

### Conflict of interest statement

The authors declare that the research was conducted in the absence of any commercial or financial relationships that could be construed as a potential conflict of interest.
